# On the Treatment of Missing Item Responses in Educational Large-Scale Assessment Data: An Illustrative Simulation Study and a Case Study Using PISA 2018 Mathematics Data

**DOI:** 10.3390/ejihpe11040117

**Published:** 2021-12-14

**Authors:** Alexander Robitzsch

**Affiliations:** 1 IPN—Leibniz Institute for Science and Mathematics Education, University of Kiel, Olshausenstraße 62, 24118 Kiel, Germany; robitzsch@leibniz-ipn.de; 2 Centre for International Student Assessment (ZIB), University of Kiel, Olshausenstraße 62, 24118 Kiel, Germany

**Keywords:** missing item responses, multiple imputation, item response model, PISA, country comparisons, Mislevy-Wu model, latent ignorability, nonignorable item responses

## Abstract

Missing item responses are prevalent in educational large-scale assessment studies such as the programme for international student assessment (PISA). The current operational practice scores missing item responses as wrong, but several psychometricians have advocated for a model-based treatment based on latent ignorability assumption. In this approach, item responses and response indicators are jointly modeled conditional on a latent ability and a latent response propensity variable. Alternatively, imputation-based approaches can be used. The latent ignorability assumption is weakened in the Mislevy-Wu model that characterizes a nonignorable missingness mechanism and allows the missingness of an item to depend on the item itself. The scoring of missing item responses as wrong and the latent ignorable model are submodels of the Mislevy-Wu model. In an illustrative simulation study, it is shown that the Mislevy-Wu model provides unbiased model parameters. Moreover, the simulation replicates the finding from various simulation studies from the literature that scoring missing item responses as wrong provides biased estimates if the latent ignorability assumption holds in the data-generating model. However, if missing item responses are generated such that they can only be generated from incorrect item responses, applying an item response model that relies on latent ignorability results in biased estimates. The Mislevy-Wu model guarantees unbiased parameter estimates if the more general Mislevy-Wu model holds in the data-generating model. In addition, this article uses the PISA 2018 mathematics dataset as a case study to investigate the consequences of different missing data treatments on country means and country standard deviations. Obtained country means and country standard deviations can substantially differ for the different scaling models. In contrast to previous statements in the literature, the scoring of missing item responses as incorrect provided a better model fit than a latent ignorable model for most countries. Furthermore, the dependence of the missingness of an item from the item itself after conditioning on the latent response propensity was much more pronounced for constructed-response items than for multiple-choice items. As a consequence, scaling models that presuppose latent ignorability should be refused from two perspectives. First, the Mislevy-Wu model is preferred over the latent ignorable model for reasons of model fit. Second, in the discussion section, we argue that model fit should only play a minor role in choosing psychometric models in large-scale assessment studies because validity aspects are most relevant. Missing data treatments that countries can simply manipulate (and, hence, their students) result in unfair country comparisons.

## 1. Introduction

It has frequently been argued that measured student performance in educational large-scale assessment (LSA; [1,2,3]) studies is affected by test-taking strategies. In a recent paper that was published in the highly ranked *Science* journal, researchers Steffi Pohl, Esther Ulitzsch and Matthias von Davier [4] argue that “current reporting practices, however, they confound differences in test-taking behavior (such as working speed and item nonresponse) with differences in competencies (ability). Furthermore, they do so in a different way for different examinees, threatening the fairness of country comparisons” [4]. Hence, the reported student performance (or, equivalently, student ability) is regarded by the authors as a conflated composite of a “true” ability and test-taking strategies. Importantly, Pohl et al. [4] question the validity of country comparisons that are currently reported in LSA studies and argue for an approach that separates test-taking behavior (i.e., item response propensity and working speed) from a purified ability measure. The core idea of the Pohl et al. [4] approach is on how to model missing item responses in educational large-scale assessment studies. In this article, we systematically investigate the consequences of different treatments of missing item responses in the programme for international student assessment (PISA) study conducted in 2018. Note that we do not focus on exploring or modeling test-taking strategies in this article.

While the treatment of missing data in statistical analyses in social sciences is now widely used [5,6,7,8], in recent literature, there are recommendations for treating missing item responses in item response theory (IRT; [9]) models in LSA studies [10,11]. Typically, the treatment of item responses can be distinguished between calibration (computation of item parameters) and scaling (computation of ability distributions).

It is essential to distinguish the type of missing item responses. Missing item responses at the end of the test are referred to as not reached items, while missing items within the test are denoted as omitted items [12]. Since the PISA 2015 study, not reached items are no longer scored as wrong and the proportion of not reached items is used as a predictor in the latent background model [13]. Items that are not administered to students in test booklets in a multiple-matrix design [13,14,15] lead to missingness completely at random (except in multi-stage adaptive testing; see [16]). This kind of missingness is not the topic of this article and typically does not cause issues in estimating population and item parameters.

Several psychometricians have repeatedly argued that missing item responses should never be scored as wrong because such a treatment would produce biased item parameter estimates and unfair country rankings [4,10,11,17,18]. In contrast, model-based treatments of missing item responses that rely on latent ignorability [4,10,11,19] are advocated. Missing item responses can be ignored in this approach when including response indicators and a latent response propensity [20,21]. Importantly, the missingness process is summarized by the latent response variable. As an alternative, multiple imputation at the level of items can be employed to handle missing item responses properly [22,23]. However, scoring missing item responses as wrong could be defended for validity reasons [24,25,26]. Moreover, it has been occasionally argued that simulation studies cannot provide information on the proper treatment of missing item responses in a concrete empirical application because the truth is unknown that would have generated the data [25,27]. Nevertheless, simulation studies could be tremendously helpful in understanding and comparing competitive statistical modeling approaches.

Our findings might only be generalizable to other low-stakes assessment studies like PISA [28,29,30]. However, the underlying mechanisms for missing item responses can strongly differ from high-stakes assessment studies [31].

Although several proposals of using alternative scaling models for abilities in LSA studies like PISA have been made, previous work either did not report country means in the metric of interest [10] such that consequences cannot be interpreted, or constituted only a toy analysis consisting only a few countries [4] that did enable a generalization to operational practice. Therefore, this article compares different scaling models that rely on different treatments of missing item responses. We use the PISA 2018 mathematics dataset as a showcase. We particularly contrast the scoring of missing item responses as wrong with model-based approaches that rely on latent ignorability [4,10,11] and a more flexible Mislevy-Wu model [32,33] containing the former two models as submodels. In the framework of the Mislevy-Wu model, it is tested whether the scoring of missing item responses as wrong or treating them as latent ignorable are preferred in terms of model fit. Moreover, it is studied whether the probability of responding to an item depends on the item response itself (i.e., nonignorable missingness, [7]). In the most general model, the missingness process is assumed to be item format-specific. Finally, we investigate the variability across means from different models for a country.

The rest of the article is structured as follows. In Section 2, an overview of different statistical modeling approaches for handling missing item responses is presented. Section 3 contains an illustrative simulation study that demonstrates the distinguishing features of the different modeling approaches. In Section 4, the sample of persons and items and the analysis strategy for the PISA 2018 mathematics case study are described. In Section 5, the results of PISA 2018 mathematics are presented. Finally, the paper closes with a discussion in Section 6.

## 2. Statistical Models for Handling Missing Item Responses

In this section, different statistical approaches for handling missing item responses are discussed. These different approaches are utilized in the illustrative simulation study (see Section 3) and the empirical case study involving PISA 2018 mathematics data (see Section 4).

For simplicity, we only consider the case of dichotomous items. The case of polytomous items only requires more notation for the description of models but does not change the general reasoning elaborated for dichotomous items. Let $X_{pi}$ denote the dichotomous item responses and the $R_{pi}$ response indicators for person *p* and item *i*. The response indicator $R_{pi}$ takes the value one if $X_{pi}$ is observed and zero if $X_{pi}$ is missing. Consistent with the operational practice since PISA 2015, the two-parameter logistic (2PL) model [34] is used for scaling item responses [13,16]. The item response function is given as

(1) $$P\left(X_{pi}=1|\theta_{p}\right)=\Psi \left(a_{i}\left(\theta_{p}-b_{i}\right)\right),$$

where Ψ denotes the logistic distribution function. The item parameters $a_{i}$ and $b_{i}$ are item discriminations and difficulties, respectively. It holds that 1−Ψ(x)=Ψ(−x). Local independence of item responses is posed; that is, item responses $X_{pi}$ are conditionally independent from each other given the ability variable $\theta_{p}$. The latent ability $\theta_{p}$ follows a standard normal distribution. If all item parameters are estimated, the mean of the ability distribution is fixed to zero and the standard deviation is fixed to one. The one-parameter logistic (1PL, [35]) model is obtained if all item discriminations are set equal to each other.

**Figure 1:**
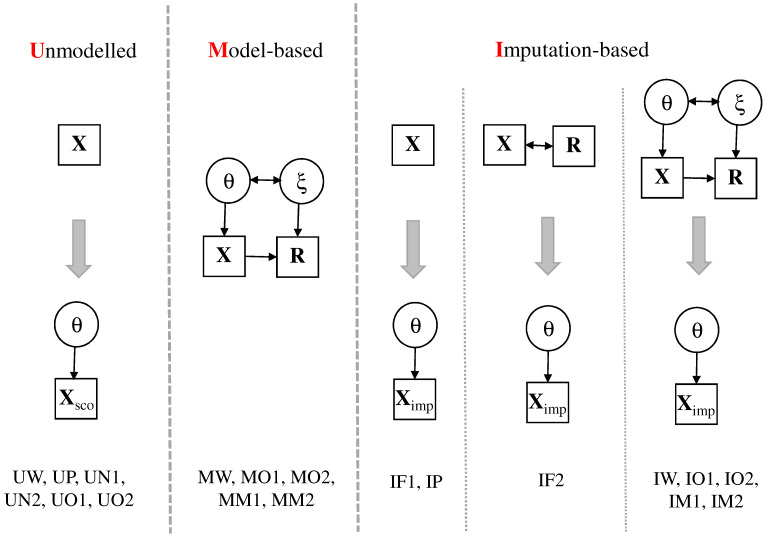
Overview of different statistical models for the treatment of missing item responses. The abbreviations of the different modeling strategies (“U”, “M” and “I”) are printed in red.

In Figure 1, the main distinctive features of the different missing data treatments are shown. Three primary strategies can be distinguished [36,37]. These strategies differ in how to include information from the response indicator variables.

First, response indicators $R_{p}$ are unmodelled (using model labels starting with “U”), and missing entries in item responses $X_{p}$ are scored using some a priorily defined rule resulting in item responses $X_{\mathrm{sco},p}$ without missing entries. For example, missing item responses can be scored as wrong or can be omitted in the estimation of the scaling model. In a second step, the 2PL scaling model is applied to the dataset containing scored item responses $X_{\mathrm{sco},p}$.

Second, model-based approaches (using model labels starting with “M”) pose a joint IRT model for item responses $X_{p}$ and response indicators $R_{p}$ [19]. The 2PL scaling model for the one-dimensional ability variable $\theta_{p}$ is part of this model. In addition, a further latent variable $\xi_{p}$ (i.e., the so-called response propensity) is included that describes the correlational structure underlying the response indicators $R_{p}$. In most approaches discussed in the literature, there is no path from $X_{pi}$ to $R_{pi}$. After controlling for ability $\theta_{p}$ and response propensity $\xi_{p}$, there is no modeled effect of the item response on the response indicator. In this paper, we allow for this additional relation by using the Mislevy-Wu model and empirically demonstrate that missingness on items depends on the item response itself.

Third, imputation-based approaches (using model labels starting with “I”) first generate multiply imputed datasets and fit the 2PL scaling model to the imputed datasets in a second step [37,38]. Different imputation models can be employed. One can either use only the item responses $X_{p}$ or use the item responses $X_{p}$ and the response indicators $R_{p}$ in the imputation model. As an alternative, imputations can be generated based on an IRT model that contains item responses $X_{p}$ and missing indicators $R_{p}$. These imputation models can coincide with IRT models that are employed as model-based approaches in our overview. After fitting the IRT models for $\left(X_{p},R_{p}\right)$, the output contains a posterior distribution $P(\theta_{p},\xi_{p}|X_{p},R_{p})$ for each subject *p*. For each imputed dataset, one first simulates latent variables $\theta_{p}^{*}$ and $\xi_{p}^{*}$ from the posterior distribution [39]. For items with missing item responses (i.e., $R_{pi}=0$), one can simulate scores for $X_{pi}$ according to the conditional distribution $P(X_{pi}=x|R_{pi}=0,\theta_{p}^{*},\xi_{p}^{*})$ (x=0,1). It holds that

(2) $$P\left(X_{pi}=1|R_{pi}=0,\theta_{p}^{*},\xi_{p}^{*}\right)=\frac{P\left(R_{pi}=0|X_{pi}=1,\xi_{p}^{*}\right)P\left(X_{pi}=1|\theta_{p}^{*}\right)}{\sum_{x=0}^{1}P\left(R_{pi}=0|X_{pi}=x,\xi_{p}^{*}\right)P\left(X_{pi}=x|\theta_{p}^{*}\right)}$$

The 2PL scaling model is applied to the imputed datasets $X_{\mathrm{imp},p}$ in a second step. In the analyses of this paper, we always created 5 imputed datasets to reduce the simulation error associated with the imputation. We stack the 5 multiply imputed datasets into one long dataset and applied the 2PL scaling model for the stacked dataset (see [40,41,42]). The stacking approach does not result in biased item parameter estimates [41], but resampling procedures are required for obtaining correct standard errors [40]. This article mainly focuses on differences between results from different models and does not investigate the accuracy of standard error computation methods based on resampling procedures.

In the next subsections, we describe the different models for treating missing item responses. These models differ with regards to the missingness mechanism assumptions of missing item responses. Some of the model abbreviations in Figure 1 are already mentioned in this section. Models that only appear in the case study PISA 2018 mathematics are described in Section 4.1.

### 2.1. Scoring Missing Item Responses as Wrong

In a reference model, we scored all missing item responses (omitted and not reached items) as wrong (model UW). The literature frequently argues that missing item responses should never be scored as wrong [4,10,17,43]. However, we think that the arguments against the scoring as wrong are flawed because these studies simulate missing item responses based on response probabilities that do not depend on the item itself. We think that these data-generating models are not plausible in applications (but see also [44] for a more complex missing model; [25,26]). On the other hand, one can simulate missing item responses such that missing item responses can only occur for incorrectly solved items (i.e., for items with $X_{pi}=0$). In this situation, all missing data treatments that do not score missing item responses as wrong will provide biased estimates [27].

### 2.2. Scoring Missing Item Responses as Partially Correct

Missing responses for MC items can be scored as partially correct (also known as fractional correct item responses; see [45]). The main idea is that a student could guess the MC item if he or she does not know the answer. If an item *i* has $K_{i}$ alternatives, a random guess of an item option would provide a correct response with probability $1/K_{i}$. In IRT estimation, one can weigh probabilities $P(X_{pi}=1)$ with $1/K_{i}$ and $P(X_{pi}=0)$ with $1-1/K_{i}$ [45]. This weighing implements a scoring of a missing MC item as partially correct (model UP). The maximum likelihood estimation is replaced by a pseudo-likelihood estimation that allows non-integer item responses [45]. More formally, the log-likelihood function *l* for estimating item parameters $a=(a_{1},\ldots ,a_{I})$ and $b=(b_{1},\ldots ,b_{I})$ can be written as

(3) $$l\left(a,b;X_{\mathrm{sco}}\right)=\sum_{p=1}^{N}\log\left(\int \prod_{i=1}^{I}\left\{\Psi {\left(a_{i}\left(\theta -b_{i}\right)\right)}^{x_{pi}}{\left[1-\Psi \left(a_{i}\left(\theta -b_{i}\right)\right)\right]}^{1-x_{pi}}\right\}f\left(\theta \right)\;d\theta \right),$$

where *f* denotes the density of the standard normal distribution, and *N* denotes the sample size. The entries $x_{pi}$ in the vector of scored item responses $X_{p}$ can generally take values between 0 and 1. The EM algorithm typically used in estimating IRT models [46,47] only needs to be slightly modified for handling fractionally correct item responses. In the M-step for computing expected counts, one must utilize the fractional item responses instead of using only zero or one values. The estimation can be carried out in the R [48] package sirt [49] (i.e., using the function rasch.mml2()).

It should be mentioned pseudo-likelihood estimation of IRT models that allow non-integer item responses is not widely implemented in IRT software. However, the partially correct scoring can be alternatively implemented by employing a multiple imputation approach of item responses. For every missing item response of item *i*, a correct item response is imputed with probability $1/K_{i}$. No imputation algorithm is required because only random guessing is assumed. This means that the guessing probability of $1/K_{i}$ is constant for persons and items.

Missing item responses for CR items are scored as wrong in the partially correct scoring approach because students in this situation cannot simply guess unknown answers.

### 2.3. Treating Missing Item Responses as Ignorable

As an alternative to scoring missing item responses as wrong, missing item responses can be ignored in likelihood estimation. In model UO1, all missing item responses are ignored in the scaling model. The student ability $\theta_{p}$ is extracted based on the observed item responses only. The log-likelihood function *l* for this model can be written as

(4) $$l\left(a,b;X,R\right)=\sum_{p=1}^{N}\log\left(\int \prod_{i=1}^{I}\left\{\Psi {\left(a_{i}\left(\theta -b_{i}\right)\right)}^{r_{pi}x_{pi}}{\left[1-\Psi \left(a_{i}\left(\theta -b_{i}\right)\right)\right]}^{r_{pi}\left(1-x_{pi}\right)}\right\}f\left(\theta \right)\;d\theta \right).$$

It can be seen from Equation (4) that only observations with observed item responses (i.e., $r_{pi}=1$) contribute to the likelihood function.

The method UO1 is valid if missing item responses can be regarded as ignorable [18]. If $X_{\mathrm{com},p}=\left(X_{\mathrm{obs},p},X_{\mathrm{mis},p}\right)$ is a partitioning of the vector of complete item responses into the observed and the missing part, the assumption that item responses are missing at random [7] is given as

(5) $$P\left(R_{p}|X_{\mathrm{obs},p},X_{\mathrm{mis},p}\right)=P\left(R_{p}|X_{\mathrm{obs},p}\right).$$

This means that the probability of omitting items only depends on observed items and not the unobserved item responses. By integrating out missing item responses $X_{\mathrm{mis},p}$, the joint distribution $\left(X_{\mathrm{com},p},R_{p}\right)$ and using the MAR assumption (5) can be written as

(6) $$\int P\left(X_{\mathrm{obs},p},X_{\mathrm{mis},p},R_{p}\right)\;dX_{\mathrm{mis},p}=P\left(R_{p}|X_{\mathrm{obs},p}\right)P\left(X_{\mathrm{obs},p}\right).$$

Hence, Equation (6) shows that likelihood inference for MAR data can entirely rely on the probability distribution $P(X_{\mathrm{obs},p})$ of observed item responses. The notion of (manifest) ignorability means that model parameters of the distributions $P(X_{\mathrm{obs},p})$ and $P(R_{p}|X_{\mathrm{obs},p})$ are distinctive. This means that these distributions can be modeled independently.

It should be emphasized that the MAR assumption (5) does not involve the latent ability $\theta_{p}$. The probability of missingness must be inferred by (summaries of) observed item responses only. This kind of missingness process might be violated in practice. In the following subsection, a weakened version of ignorability is discussed.

### 2.4. Treating Missing Item Responses as Latent Ignorable

Latent ignorability [19,50,51,52,53,54,55,56,57,58,59,60] is one of the weakest nonignorable missingness mechanisms. Latent ignorability weakens the assumption of ignorability for MAR data. In this case, the existence of a latent variable $\eta_{p}$ is assumed. The dimension of $\eta_{p}$ is typically much lower than the dimension of $X_{p}$. Latent ignorability is defined as (see [19])

(7) $$P\left(R_{p}|X_{\mathrm{obs},p},X_{\mathrm{mis},p},\eta_{p}\right)=P\left(R_{p}|X_{\mathrm{obs},p},\eta_{p}\right).$$

That is, the probability of missing item responses depends on observed item responses and the latent variable $\eta_{p}$, but not the unknown missing item responses $X_{\mathrm{mis},p}$ itself. By integrating out $X_{\mathrm{mis},p}$, we obtain

(8) $$\int P\left(R_{p},X_{\mathrm{obs},p},X_{\mathrm{mis},p}|\eta_{p}\right)\;dX_{\mathrm{mis},p}=P\left(R_{p}|X_{\mathrm{obs},p},\eta_{p}\right)P\left(X_{\mathrm{obs},p}|\eta_{p}\right).$$

The specification (7) is also known as a shared-parameter model [61,62]. In most applications, conditional independence of item responses $X_{pi}$ and response indicators $R_{pi}$ conditional on $\eta_{p}$ is assumed [19]. In this case, Equation (8) simplifies to

(9) $$\int P\left(R_{p}=r_{p},X_{\mathrm{obs},p}=x_{\mathrm{obs},p},X_{\mathrm{mis},p}|\eta_{p}\right)\;dX_{\mathrm{mis},p}=\prod_{i=1}^{I}\left[P\left(R_{pi}=r_{pi}|\eta_{p}\right)P{\left(X_{pi}=x_{pi}|\eta_{p}\right)}^{r_{pi}}\right].$$

In the rest of this paper, it is assumed that the latent variable $\eta_{p}$ consists of a latent ability $\theta_{p}$ and a latent response propensity $\xi_{p}$. The latent response propensity $\xi_{p}$ is a unidimensional latent variable that represents the dimensional structure of the response indicators $R_{p}$. The probability of responding to an item is given by (model MO2; [10,20,44,63,64,65,66])

(10) $$P\left(R_{pi}=1|X_{pi}=x_{pi},\theta_{p},\xi_{p}\right)=P(R_{pi}=1|\xi_{p})=\Psi \left(\xi_{p}-\beta_{i}\right).$$

Note that the probability of responding to item *i* only depends on $\xi_{p}$ and is independent of $X_{pi}$ and $\theta_{p}$. The 2PL model is assumed for item responses $X_{pi}$ (see Equation (1)):

(11) $$P\left(X_{pi}=1|\theta_{p},\xi_{p}\right)=P\left(X_{pi}=1|\theta_{p}\right)=\Psi \left(a_{i}\left(\theta_{p}-b_{i}\right)\right).$$

The model defined by Equations (10) and (11) is also referred to as the Holman–Glas model [20,37]. In this article, a bivariate normal distribution for $\left(\theta_{p},\xi_{p}\right)$ is assumed, where $\mathrm{SD}(\theta_{p})$ is fixed to one, and $\mathrm{SD}(\xi_{p})$, as well as $\mathrm{Cor}(\theta_{p},\xi_{p})$, are estimated (see [67,68] for more complex distributions).

The model UO1 (see Section 2.3) that presupposes ignorability (instead of latent ignorability) can be tested as a nested model within model MO2 by setting $\mathrm{Cor}(\theta_{p},\xi_{p})=0$. This model is referred to as model MO1.

Note that the joint measurement model for item responses $X_{pi}$ and response indicators $R_{pi}$ can be written as

(12) $$P\left(X_{pi}=x,R_{pi}=r|\theta_{p},\xi_{p}\right)=\left\{\begin{array}{cc} \left[1-\Psi (a_{i}\left(\theta_{p}-b_{i}\right))\right]\Psi \left(\xi_{p}-\beta_{i}\right) & \mathrm{if}\;x=0\;\mathrm{and}\;r=1,\\ \Psi (a_{i}\left(\theta_{p}-b_{i}\right))\Psi \left(\xi_{p}-\beta_{i}\right) & \mathrm{if}\;x=1\;\mathrm{and}\;r=1,\\ 1-\Psi (\xi_{p}-\beta_{i}) & \mathrm{if}\;x=\mathrm{NA}\;\mathrm{and}\;r=0. \end{array}\right.$$

Hence, the model defined in Equation (12) can be interpreted as an IRT model for a variable $V_{pi}$ that has three categories: Category 0 (observed incorrect): $X_{pi}=0$, $R_{pi}=1$, Category 1 (observed correct): $X_{pi}=1$, $R_{pi}=1$, and Category 2 (missing item response): $X_{pi}=\mathrm{NA}$, $R_{pi}=0$ (see [43,69,70]).

#### 2.4.1. Generating Imputations from IRT Models Assuming Latent Ignorability

The IRT models MO1 and MO2 are also used for generating multiply imputed datasets. Conditional on $\theta_{p}$, missing item responses are imputed according to the response probability from the 2PL model (see Equation (11)). The stacked imputed dataset is scaled with the unidimensional 2PL model. If models MO1 or MO2 were be the true data-generating models, the results from multiple imputation (i.e., IO1 and IO2) would coincide with model-based treatments (i.e., MO1 and MO2). However, results can differ in the case of misspecified models [71,72].

#### 2.4.2. Including Summaries of Response Indicators in the Latent Background Model

The IRT model for response indicators $R_{pi}$ in Equation (10) is a 1PL model. Hence, the sum score $R_{p•}=\sum_{i=1}^{I}R_{pi}$ is a sufficient statistic for the response propensity $\xi_{p}$ [73]. Then, the joint distribution can be written as

(13) $$P\left(R_{p},X_{\mathrm{obs},p},\theta_{p},\xi_{p}\right)P\left(\theta_{p}|\xi_{p}\right)P\left(\xi_{p}\right)=P\left(X_{\mathrm{obs},p}|\theta_{p}\right)\left\{P\left(\theta_{p}|\xi_{p}\right)P\left(R_{p•}|\xi_{p}\right)P\left(\xi_{p}\right)\right\}.$$

Instead of estimating a joint distribution $\left(\theta_{p},\xi_{p}\right)$, a conditional distribution $\theta_{p}|R_{p•}$ can be specified in a latent background model (LBM; [74,75]). That is, one uses the proportion of missing item responses $Z_{p}=1-R_{p•}/I$ as a predictor for $\theta_{p}$ [11,12] and employs a conditional normal distribution $\theta_{p}|Z_{p}∼N\left(\gamma_{0}+\gamma_{1}Z_{p},\sigma_{e}^{2}\right)$. This manifest variable $Z_{p}$ can be regarded as a proxy variable for the latent variable $\xi_{p}$. The resulting model is referred to as model UO2.

### 2.5. Mislevy-Wu Model for Nonignorable Item Responses

Latent ignorability characterizes only a weak deviation from an ignorable missing data process. It might be more plausible that the probability $P(R_{pi}=1|X_{pi},\theta_{p},\xi_{p})$ of responding to an item depends on the observed or unobserved item response $X_{pi}$ itself [76,77,78,79,80]. The so-called Mislevy-Wu model [32,33,81,82] extends the model MO2 (see Equation (10)) that assumes latent ignorability to

(14) $$P\left(R_{pi}=1|X_{pi},\theta_{p},\xi_{p}\right)=\Psi \left(\xi_{p}-\beta_{i}-\delta_{i}X_{pi}\right).$$

In this model, the probability of responding to an item depends on the latent response propensity $\xi_{p}$ and the item response $X_{pi}$ itself (see [24,25,49,81,83,84]). The parameter $\beta_{i}$ governs the missingness proportion for $X_{pi}$ in the subgroup of persons with $X_{pi}=0$, while the sum $\beta_{i}+\delta_{i}$ represents the missingness proportion for persons with $X_{pi}=1$. The unique feature of the Mislevy-Wu model is that the missingness proportion is allowed to depend on the item response. If a very small negative value for the missingness parameter $\delta_{i}$ is chosen (e.g., $\delta_{i}=-10$), the response probability $P(R_{pi}=1|X_{pi},\theta_{p},\xi_{p})$ in Equation (14) is close to one, meaning that persons with $X_{pi}=1$ always provide item response (i.e., they have a missing proportion of zero). By applying the Bayes theorem, it follows in this case that persons with a missing item response must possess an incorrectly solved item; that is, it holds $X_{pi}=0$. It should be emphasized that the Mislevy-Wu model is a special case of models discussed in [85].

Model MM1 is defined by assuming a common $\delta_{i}$ parameter for all items. In model MM2, two δ parameters are estimated for item formats CR and MC in the PISA 2018 mathematics case study (see Section 5 for results).

Note that the Mislevy-Wu model for item responses $X_{pi}$ and response indicators $R_{pi}$ can be also formulated as a joint measurement model for a polytomous item with three categories 0 (observed incorrect), 1 (observed correct), and 2 (missing; see also Equation (12)):

(15) $$P\left(X_{pi}=x,R_{pi}=r|\theta_{p},\xi_{p}\right)=\left\{\begin{array}{cc} \left[1-\Psi (a_{i}\left(\theta_{p}-b_{i}\right))\right]\Psi \left(\xi_{p}-\beta_{i}\right) & \mathrm{if}\;x=0\;\mathrm{and}\;r=1,\\ \Psi (a_{i}\left(\theta_{p}-b_{i}\right))\Psi \left(\xi_{p}-\beta_{i}-\rho_{i}\right) & \mathrm{if}\;x=1\;\mathrm{and}\;r=1,\\ \Psi (a_{i}\left(\theta_{p}-b_{i}\right))\Psi \left(\xi_{p}-\beta_{i}-\rho_{i}\right)+\left[1-\Psi (a_{i}\left(\theta_{p}-b_{i}\right))\right]\Psi \left(\xi_{p}-\beta_{i}\right)\; & \mathrm{if}\;x=\mathrm{NA}\;\mathrm{and}\;r=0. \end{array}\right.$$

The most salient property of the models MM1 and MM2 is that the model treating missing item responses as wrong (model UW) can be tested by setting $\delta_{i}=-10$ in Equation (14) (see [33]). This model is referred to as model MW and the corresponding scaling model based on multiply imputed datasets from MW as model IW. Moreover, the model MO2 assuming latent ignorability is obtained by setting $\delta_{i}=0$ for all items *i* (see Equation (10)). It has been shown that parameter estimation in the Mislevy-Wu model and model selection among models MW, MO2, and MM1 based on information criteria have satisfactory performance [33].

For both models, multiply imputed datasets were also created based on conditional distributions $P(X_{pi}|R_{pi},\theta_{p},\xi_{p})$. The scaling models based on stacked imputed datasets are referred to as IM1 and IM2.

### 2.6. Imputation Models Based on Fully Conditional Specification

The imputation models discussed in previous subsections are based on unidimensional or two-dimensional IRT models (see [36,86,87,88,89] for more imputation approaches relying on strong assumptions). Posing such a strict dimensionality assumption might result in invalid imputations because almost all IRT models in educational large-scale assessment studies are likely to be misspecified [26]. Hence, alternative imputation models for missing item responses were considered that relied on fully conditional specification (FCS; [41]) implemented in the R package mice [90].

The FCS imputation algorithm operates as follows (see [41,91,92,93]). Let $W_{p}$ denote the vector of variables that can have missing values. FCS cycles through all variables in $W_{p}$ (see [37,94,95,96]). For variable $W_{pv}$, all remaining variables in $W_{p}$ except $W_{pv}$ are used as predictors for $W_{pv}$ (denotes as $W_{p,(-v)}$) in the imputation model. More formally, a linear regression model

(16) $$W_{pv}=\gamma_{0}+\gamma^{⊤}W_{p,(-v)}+\varepsilon_{pv}\;,\;\varepsilon_{pv}∼N\left(0,\sigma_{v}^{2}\right)$$

is specified. For dichotomous variables $W_{pv}$, (16) might be replaced by a logistic regression model. Our experiences correspond with those from the literature that using a linear regression with predictive mean matching (PMM; [41,97,98,99]) provides more stable estimates of the conditional imputation models. PMM guarantees that imputed values only take values that are present in the observed data (i.e., values of 0 or 1 for dichotomous item responses).

In situations with many items, $W_{p,(-v)}$ is a high-dimensional vector of covariates in the imputation model (16). To provide a stable and efficient estimation of the imputation model, a dimension reduction method for the vector of covariates can be applied to enable a feasible estimation. For example, principal component analysis [100] or sufficient dimension reduction [101] can be applied in each imputation model for reducing the dimensionality of $W_{p,\left(-v\right)}$. In this paper, partial least squares (PLS) regression [102] is used for transforming the vector of covariates to a low-dimensional vector of PLS factors that successively maximize the covariance with the criterion variable (i.e., maximize the covariance $\mathrm{Cov}(\alpha_{f}^{⊤}W_{p,(-v)},W_{pv})$ with factor loading vectors $\alpha_{f}$ for uncorrelated factors $\alpha_{f}^{⊤}W_{p,(-v)}$ with f=1,…,F; see [103]). In the simulation study and the empirical case study, we use 10 PLS factors to avoid the curse of dimensionality due to estimating too many parameters in the regression models [103,104].

In the imputation model IF1, only item responses $X_{p}$ are included. This specification will provide approximately unbiased estimates if the MAR assumption (i.e., manifest ignorability) holds. In model IF2, response indicators $R_{p}$ are additionally included [105]. This approach is close to the assumption of latent ignorability in which summaries of the response indicators are also required for predicting the missingness of an item response. Hence, it can be expected that the model IF2 outperforms IF1 and provides similar results to the model MO2 relying on latent ignorability. In contrast to the Mislevy-Wu model, for imputing item response $X_{pi}$ in model IF2, the predictors $X_{p,-(i)}$ and $R_{p,(-i)}$ are used. Hence, the probability of responding to an item is not allowed to depend on the item itself. This assumption might be less plausible than assuming the response model in Equation (14).

Like for all imputation-based approaches in this paper, 5 multiply imputed datasets were created, and the 2PL scaling model is applied to the stacked dataset involving all imputed datasets.

## 3. Illustrative Simulation Study

In order to better understand the relations between different models for the treatment of missing item responses, we performed a small illustrative simulation study to provide insights into the behavior of the most important models under a variety of data-generating models.

### 3.1. Method

We restrict ourselves to the analysis of only one group. This does not imply interpretational issues because the main motivation of this study is to provide a better insight into the behavior of the models and not to mimic the PISA application involving 45 countries. We only employed a fixed number of I=20 items in a linear fixed test design. Hence, we did not utilize a multi-matrix design with random allocation of students to test booklets as implemented in PISA. In our experience, we have not (yet) seen any simulation study whose results with a multi-matrix test design substantially differ from a linear fixed test design. We chose a sample size of N=1500, which corresponds to a typical sample size at the item level in the PISA application.

Item responses were generated based on the Mislevy-Wu model (see Equation (10)). Item responses were simulated according to the 2PL model. We fixed the correlation of the latent ability θ and the latent response propensity ξ to 0.5. We assumed item difficulties that were equidistantly chosen on the interval [−2,2] (i.e., −2.000, −1.789, −1.579, …, 1.789, 2.000), and we used item discriminations of 1 when simulating data. The ability variable θ was assumed to be standard normally distributed. For the response mechanism in the Mislevy-Wu model in Equation (10), we varied a common missingness parameter δ in five factor levels −10, −3, −2, −1, and 0. The case δ=−10 effectively corresponds to the situation in which missing item responses can only be produced by incorrect item responses. This simulation condition refers to the situation in which missing item responses must be scored as wrong for obtaining unbiased statistical inference. The situation δ=0 corresponds to the situation of latent ignorability. The cases δ=−3,−2,−1 correspond to situations in which both the scoring as wrong and latent ignorability missing data treatment are not consistent with the data-generating model, and biased estimation can be expected. For the model for response indicators, we used a common β parameter across items in the simulation. As our motivation was to vary the average proportion of missing item responses (i.e., the factor levels were 5%, 10%, 20%, and 30%), the common β parameter is a function of the δ parameter. Prior to the main illustrative simulation, we numerically determined the β parameter to obtain the desired missing data proportion rate (see Table A1 in Appendix A for the specific values used).

Seven analysis models were utilized in this simulation study. First, we evaluated the performance of the 2PL model for complete data (model CD). Second, we estimated the Mislevy-Wu model assuming a common missingness parameter δ (model MM1; Section 2.5). Third, we applied the method of scoring of missing items as wrong in model UW. Fourth, in contrast to UW, missing item responses were ignored in the estimation in model UO (Section 2.3). Fifth, we estimated the model with response propensity ξ relying on latent ignorability (model MO2, Section 2.4). Furthermore, two imputation-based approaches were used that rely on the fully conditional specification approach implemented in the R package mice [90]. For both approaches, five multiply imputed datasets were utilized, and the 2PL models were estimated by using a stacked dataset containing all five imputed datasets. Sixth, the model IF1 uses item responses in the imputation approach that employs PMM. Seventh, the model IF2 uses item responses and response indicators in the imputation model. To avoid multicollinearity issues, PLS imputation with 10 PLS factors was applied for models IF1 and IF2.

The 2PL analysis models provided item difficulties and item discriminations and fixed the ability distribution to the standard normal distribution. To enable a comparison of the estimated mean and the standard deviation with the mean and the standard deviation of the data-generating model, estimated item parameters were linked to the true item parameters used in the data-generating model. As a result, a mean and a standard deviation as a result of the linking procedure is compared to the true mean (i.e., M = 0) and the true standard deviation (SD = 1). In this simulation, we applied Haberman linking [106,107] that is equivalent to log-mean-mean linking for two groups [108]. Note that we use Haberman linking for multiple groups (i.e., multiple countries) in the case study in Section 4.

A total number of 500 replications was carried out for each cell of the design. We evaluated bias and root mean square error (RMSE) for the estimated mean and standard deviation. We also assessed Monte Carlo standard errors for bias, and RMSE are calculated based on the jackknife procedure [109,110]. Twenty jackknife zones were defined for the computing of the Monte Carlo standard errors.

In this illustrative simulation study, the statistical software R [48] along with the packages mice [90] and sirt [49] are used.

### 3.2. Results

In Table 1, the bias for the mean and the standard deviation for different missing data treatments as a function of the missing proportion and the missingness parameter δ is shown. In the case of complete data (CD), no biases exist. Except for the situation of a large proportion of missing item responses of 30% and an extreme δ parameter of −10 (bias = 0.054), the Mislevy-Model (model MM1)—that is consistent with the data-generating model—performed very well in terms of bias for the mean and the standard deviation. If missing data were only caused by wrong items (i.e., δ=−10), models that rely on ignorability (UO, IF1) or latent ignorability (MO2, IF2) produced large biases (e.g., for the mean in the condition of 10% missing data UO 0.159, MO2 0.149, IF1 0.160, IF2 0.152). As was to be expected in this case, scoring missing item responses as wrong provided unbiased results. In contrast, if the data-generating model relied on latent ignorability (i.e., δ=0), scoring missing item responses as wrong provided biased estimates (e.g., for the mean for 10% missing data, the bias was −0.139). Note that in this condition, MO2 and IF2 provided unbiased estimates, while the models that did not take response indicators into account provided biased estimates (e.g., for the mean for 10% missing data: UO 0.037, IF1 0.038).

For values of the missingness parameter δ between −10 and 0, both missing data treatments as wrong and latent ignorable provided biased estimates for the mean. The biases were much more pronounced for higher missing data proportions. Moreover, the standard estimation is substantially underestimated when relying on a model for latent ignorability if the latent ignorability was not used for simulating item responses. Interestingly, the imputation model IF2 that uses both item responses and response indicators showed similar behavior to the model MO2 that involves the latent response propensity ξ, while the imputation model IF1 only using item responses performed similarly to UO. The standard deviation was underestimated in many conditions for the models assuming latent ignorability if the Mislevy-Wu model holds.

The Monte Carlo standard errors for the bias of the mean (M = 0.0023, SD = 0.0005, Max = 0.0044) were similar to those of the standard deviation (M = 0.0022, SD = 0.0005, Max = 0.0038). The uncertainty in the bias estimates is negligible to the variation across different missing data treatments. Hence, the conclusions obtained from this simulation study can be considered trustworthy.

In Table A2 in Appendix A, the RMSE for the mean and the standard deviation for the different missing data treatments are shown as a function of the missing data proportion and the missingness parameter δ. In situations where the models UW or MO2 provided unbiased estimates, the Mislevy-Wu model MM1 has slightly larger variable estimates. However, only in these particular situations, the RMSE of the simpler restrictive models was smaller than those of MM1. In general situations, the increase in variability was outperformed by a lower bias of model MM1. The Monte Carlo standard error for the RMSE of the mean was on average 0.0023 (SD = 0.0006, Max = 0.0044). The corresponding Monte Carlo error for the RMSE of the standard deviation turned out to be quite similar (M = 0.0023, SD = 0.0007, Max = 0.0042).

### 3.3. Summary

In this illustrative simulative study, we showed that one could not generally conclude that missing items must never be scored wrong. Moreover, models that treat missing item responses as latent ignorable do not guarantee a smaller bias compared to the scoring as wrong. In general, the scoring as wrong can provide negatively biased mean estimates, while the treatment as latent ignorable will typically provide positively biased estimates.

As with any simulation study, the data-generating truth must be known in advance which is not the case in any empirical application. The Mislevy-Wu model is a general model for treating nonignorable missing item responses. It certainly has the potential to provide less biased estimates than alternatives recently discussed in the literature.

## 4. PISA 2018 Mathematics Case Study: Method

### 4.1. Sample

The mathematics test in PISA 2018 [16] was used to investigate different treatments of missing item responses. We included 45 countries that did receive the main test in a computer-based administration. These countries did not receive test booklets with items of lower difficulty that were included for low-performing countries.

In total, 72 test booklets were administered in the computer-based assessment in PISA 2018 [16]. Test booklets were compiled from four clusters of items of the same ability domain (i.e., mathematics, reading, science). We selected only booklets which had two item clusters of mathematics items. We took booklets from students that had two item clusters containing mathematics items. Students from booklets 1 to 12 were selected. The cluster of mathematics items appeared either at the first and second (booklets 7 to 12) or the third and fourth positions (booklets 1 to 6) in the test.

As a consequence, 70 mathematics items were included in our analysis. In each of the selected booklets, 22, 23, or 24 mathematics items were administered. Seven of the 70 items were polytomous and were dichotomously recoded, with only the highest category being recoded as correct. In total, 27 out of 70 items had the complex multiple-choice (MC) format, and 43 items had constructed-response (CR) format. For 18 MC items, there were 4 response alternatives, 4 MC items had 8 response alternatives, and 5 MC items had 16 response alternatives.

In Table 2, descriptive statistics for the sample used in our analysis are presented. In total, 167,092 students from these 45 countries were included in the analysis. On average, M=3713.2 students were available in each country. The average number of students per item within each country ranged between 415.8 (MLT, Malta) and 4408.3 (ESP, Spain). On average, M=1120.3 students per item were available at the country level.

**Figure 2:**
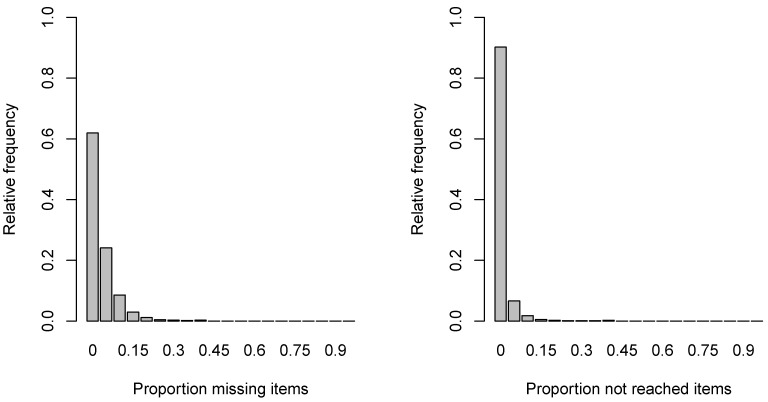
Frequency distribution of missing item responses (**left** panel) and not reached items at the student level (**right** panel).

The average proportion of missing item responses in the dataset was 8.4% (SD=3.3%) and ranged between 1.2% (MYS, Malaysia) and 18.8% (BIH; Bosnia and Herzegovina). The proportion of not reached item responses was on average 2.4% (SD=1.0%) with the maximum of 5.9% (SWE, Sweden). Interestingly, the missing data proportions and the country means were only moderately correlated (Cor=−0.48). Missing proportions for CR items were substantially larger (M=12.3%, SD=4.8%, Min=1.5%, Max=27.9%) than for MC items (M=2.3%, SD=1.0%, Min=0.7%, Max=5.4%). Figure 2 shows the distribution of the proportion of missing and not reached items at the student level aggregated across countries. Most students produced no missing items (i.e., 61.9%) or no not reached items (i.e., 90.2%).

### 4.2. Scaling Models

The different scaling models for treating missing item responses are compared for the PISA 2018 mathematics data for country means and country standard deviations. To compare the parameters of ability distributions across countries, different strategies are considered viable in the literature. These strategies will typically provide different results in the presence of differential item functioning between countries (country DIF; [111,112,113,114]). In this situation, item parameters vary across countries, they are not invariant across countries. First, the noninvariance can be ignored in the scaling model. A misspecified model assuming invariant item parameters is purposely specified [114,115,116,117,118]. Second, scaling is conducted under partial invariance in which only a portion of item parameters is allowed to differ across countries [13,16,119,120,121,122]. Third, a hierarchical model is utilized as the scaling model in which country-specific item parameters are modeled as random effects [111,123,124]. Fourth, the scaling models are separately applied for each country in the first step. In a second step, a common metric is established by applying a linking procedure that transforms item parameters and the ability distribution [108,118,125].

In our analysis, we use the linking approach relying on separate scalings for comparing the ability distribution across countries. We opted for this strategy for the following reasons. First, it is likely that the missingness mechanisms differ across countries [126]. Hence, in a model-based approach to treating missing item responses, it does not seem justified to assume invariant model parameters for the missingness mechanism across countries. Second, it has been shown in the presence of country DIF that a misspecified scaling model assuming invariant item parameters provides more biased parameter estimates than those obtained from the linking approach [127]. Third, large models that concurrently scale all countries (assuming full invariance or partial invariance) are less robust to model deviations. Fourth, we argued elsewhere that the partial invariance approach currently used in PISA results in invalid country comparisons because the comparisons of each pair of countries essentially rely on different sets of items [26,114,118]. Fifth, the linking approach is computationally much less demanding than concurrent scaling approaches (assuming invariance or partial invariance; see [118,125,128]).

As argued above, the scalings the analysis of our PISA 2018 mathematics case study are carried out separately for each country *c*. That is, one obtains country-specific item parameters $a_{ic}$ and $b_{ic}$:

(17) $$P\left(X_{pci}=1|\theta_{pc}\right)=\Psi \left(a_{ic}\left(\theta_{pc}-b_{ic}\right)\right)\;,\;\theta_{pc}∼N\left(0,1\right)\;.$$

Sampling weights were always used when applying the scaling model (17) to the PISA 2018 dataset. To enable the comparability of the ability distribution across countries, the obtained item discriminations $a_{ic}$ and item difficulties $b_{ic}$ are transformed on a common in a subsequent linking step (see Section 4.3) for details.

For the PISA 2018 mathematics data, the scaling models discussed in Section 2 are applied. An overview of the specified models with brief explanations is given in Table 3. Some of the models required particular adaptations that are described in the two following subsections.

#### 4.2.1. Treating Not Reached Items as Ignorable or in the Latent Background Model

Since PISA 2015, not reached items are no longer scored as wrong [13]. To investigate this scaling method, we ignored not reached items in the scaling model but scored omitted items as wrong (model UN1). We also implemented the operational practice since PISA 2015 [13] that includes the proportion of not reached item response as a predictor in the latent background model (model UN2; [12,129]). This second model is similar to assuming latent ignorability when the response indicators for not reached items follow a 1PL model.

#### 4.2.2. Imputation Models Based on Fully Conditional Specification

In Section 2.6, we introduced the FCS imputation models IF1 and IF2 that used $X_{p}$ and $\left(X_{p},R_{p}\right)$ in the imputation, respectively. Previous research indicated that item parameters are affected by position effects [130,131,132,133,134,135,136,137]. Hence, in our analysis, the FCS imputation models IF1 and IF2 are separately applied for each test booklet. In general, missing item responses at the end of a test booklet will be less likely imputed with a correct scoring (i.e., $X_{pi}=1$) than missing item responses at the beginning of a test booklet. As the sample size for each country in each test booklet can be quite low, using PLS regression for dimension reduction of the covariates in the imputation models is vital.

### 4.3. Linking Procedure

The scaling models described above resulted in country-specific item discriminations $a_{ic}$ and item difficulties $b_{ic}$. To enable a comparison of country means and country standard deviations, the corresponding ability distributions can be obtained by linking approaches that establish a common ability metric [108,138]. In this article, Haberman linking [107] in its original proposal is used. The linking procedure produces country means and standard deviations as its outcome. To enable a comparisons across the 19 specified different scaling models, the ability distributions were linearly transformed such that the total population involving all students in all countries in our study has a mean M=500 and a standard deviation SD=100 (i.e., the so-called PISA metric). More formally, for each model *m* and each country *c*, there is a linear transformation $\theta \mapsto t_{mc}\left(\theta \right)=\nu_{0mc}+\nu_{1mc}\theta$ that transforms the country-specific ability distributions obtained from separate scaling to the PISA metric.

### 4.4. Model Comparisons

It is of particular interest whether the Mislevy-Wu model (MM1 and MM2) outperforms other treatments of missing item responses such as the scoring as wrong (model MW) and latent ignorable (models MO1 and MO2). The Bayesian information criterion (BIC) is used for conducting model comparisons ([33]; see also [16,120,121,139] for similar model comparisons in PISA, but [140,141,142] for improved information criteria in complex surveys). Moreover, the Gilula–Haberman penalty (GHP; [143,144,145]) is used as an effect size that is relatively independent of the sample size and the number of items. The GPH is defined as $\mathrm{GHP}=\mathrm{AIC}/(2\sum_{p=1}^{N}I_{p})$, where $I_{p}$ is the number of estimated model parameters for person *p* and AIC is the Akaike information criterion. For example, if 20 out of 70 items were administered to person *p* in a test, $I_{p}$ would be 40 in the 2PL model. If a student worked on all 70 items in the test, $I_{p}$ would be 140. Note that the GHP can be considered a normalized variant of the AIC. A difference in GHP larger than 0.001 is declared a notable difference in model fit [145,146].

It might be questioned whether information criteria AIC (for the GHP criterion) and BIC might be appropriate for datasets $\left(X_{pi},R_{pi}\right)$ consisting of item responses and response indicators with missing data on item responses $X_{pi}$ (see [147,148,149,150]). As was argued in Section 1, there are two types of missing item responses in large-scale assessment datasets. First, item responses can be missing for a student because only a portion of items was administered in a test booklet in the multi-matrix test design [16]. Second, missing item responses appear due to item omissions to administered items. The latter type of missingness is the main topic of this article.

It has been demonstrated in Section 2.5 (see Equation (15)) that for each item *i*, observations $\left(X_{pi},R_{pi}\right)$ can be regarded as a random variable $V_{pi}$ with three categories: Category 0 ($V_{pi}=0$): $X_{pi}=0,R_{pi}=1$, Category 1 ($V_{pi}=1$): $X_{pi}=1,R_{pi}=1$, and Category 2 ($V_{pi}=2$): $X_{pi}=\mathrm{NA},R_{pi}=0$. The dataset with observations $V_{pi}$ does not contain missing values, and the Mislevy-Wu model can be formulated as a function of $V_{pi}$ instead of $\left(X_{pi},R_{pi}\right)$. As the former dataset does not contain missing values, model selection based on information criteria might be justified for item omissions because no missing data occurs for the redefined variables. However, it might still be questioned whether information criteria AIC and BIC remain valid when applied to multi-matrix designs. In this case, the number of effectively estimated item parameters per student is lower than those obtained when all items would be administered in a test booklet. In our opinion and our limited experience obtained in an unpublished simulation study, it could be that AIC and BIC show inferior performance for multi-matrix designs compared to the complete-data case. Note also that most educational large-scale assessment studies also apply the conventional information criteria without adaptations (e.g., [121,139,151,152,153,154]).

We would like to point out that BIC and GHP are only applied for the model-based treatment scaling models and not to the scaling models that rely on multiply imputed datasets (see [155]).

### 4.5. Computation of Standard Errors

In the PISA study, statistical inference is typically conducted with the balanced repeated replication methodology to account for stratified clustered sampling within countries [16,156]. The *r*th replication sample uses a modified set of person sampling weights $w_{p}^{\left(r\right)}$. Using R=80 replication samples in PISA, a parameter of interest is computed for the original sample (i.e., $\hat{\gamma }$) based on student weights $w_{p}$. Moreover, the analysis is repeated in each replication sample using sampling weights $w_{p}^{\left(r\right)}$, resulting in parameter estimates ${\hat{\gamma }}^{\left(r\right)}$. The standard error for $\hat{\gamma }$ is then calculated as [16]

(18) $$\mathrm{SE}\left(\hat{\gamma }\right)=\sqrt{A\sum_{r=1}^{R}{\left({\hat{\gamma }}^{\left(r\right)}-\hat{\gamma }\right)}^{2}},$$

where the scaling factor *A* equals 0.05 in the PISA replication design. In our analysis, we are interested in standard errors for country means. The standard error is first determined for the country mean obtained in country-specific scaling models. Each scaling model provides a person-specific individual posterior distribution $h_{p}\left(\theta_{t}|X_{p},R_{p}\right)$ for a discrete grid $\theta_{t}$(t=1,…,T) of θ points (e.g., for T=21 integration points, a discrete θ grid $\theta_{1}=-5,\ldots ,\theta_{21}=5$ can be chosen). These posterior distributions reflect the subject-specific uncertainty with respect to the estimated ability. The country means have to be computed in the transformed metric (see Section 4.3). Hence, one uses the transformed grid $\nu_{0mc}+\nu_{1mc}\theta_{t}$ (t=1,…,T) for determining the country mean. For the *r*th replication sample, the mean ${\hat{\gamma }}^{\left(r\right)}$ is determined as

(19) $${\hat{\gamma }}^{\left(r\right)}=\frac{\sum_{p=1}^{N}w_{p}^{\left(r\right)}\sum_{t=1}^{T}h_{p}\left(\theta_{t}|X_{p},R_{p}\right)\left(\nu_{0mc}+\nu_{1mc}\theta_{t}\right)}{\sum_{p=1}^{N}w_{p}^{\left(r\right)}}.$$

Note that this approach is a numerical approximation technique that coincides with the plausible value technique [129] when a large number of plausible values would be used. The standard error for $\hat{\gamma }$ can be computed using (18). In our analysis, we are also interested in determining the statistical inference of a difference in means for a particular country resulting from different models. It is not appropriate to compute the standard errors for the means of the different models and to apply the t-test for a mean difference relying on independent samples because two models are applied to the same dataset resulting in highly dependent parameter estimates. However, the replication technique in Equation (18) can also be applied for the difference in means. One must only compute a mean difference in each replication sample in this case.

## 5. PISA 2018 Mathematics Case Study: Results

### 5.1. Similarity of Scaling Models

Each of the 19 scaling models provided a set of country means. For each country, the absolute difference of two means of a country stemming from a pair of two models can be computed. Table 4 summarizes the average absolute differences. Scaling models that resulted in an average absolute difference of at most 1.0 can be considered similar. In Table 4, groups of models are grayed in the rectangles containing the absolute differences classified as similar. Table 4 indicates that the methods that treat missing item responses as wrong (UW, MW, IW) or treat MC items as partially correct (UP, IP) resulted in similar country mean estimates. Both methods that did not score nor reached item responses as wrong (UN1, UN2) resulted in relatively similar estimates. The models that rely on ignorability (UO1, MO1, IO1) or latent ignorability (MO2, UO2, IO2) provided similar estimates. In line with previous research [18], the inclusion of the latent response propensity ξ did not result in strongly different estimates of country means compared to models that ignore missing item responses. The specifications of the Mislevy-Wu model (MM1, IM1, MM2, IM2) resulted in similar country means. Interestingly, country means from the Mislevy-Wu model were more similar to the treatment of missing item responses as wrong than those that relied on ignorability or latent ignorability. Finally, the scaling model based on FCS imputation involving only item responses (IF1) was similar to the models assuming (latent) ignorability (UO1, MO1, IO1, MO2, UO2, IO2). FCS imputation involving item responses and response indicators different from the imputed item (IF2) were neither similar to the ignorability-based treatment nor the scoring as wrong or the Mislevy-Wu model. This finding could be explained by the fact that the imputation method IF2 is based on strongly opposing assumptions of the missingness mechanism than the Mislevy-Wu model.

### 5.2. Model Comparisons

From Table 5, we can see that for the majority of countries (35 out of 45), the IRT model treating missing item responses as wrong (model MW) provided a better model fit in terms of BIC than modeling it with a latent propensity (model MO2). For 39 out of 45 countries, the Mislevy-Wu model with item-format specific ρ parameters (model MM2) was preferred. In 5 out of 45 countries, the Mislevy-Wu model with one common ρ parameter (MM1) was the best-fitting model. Only in one country (MYS), the model treating missing item responses as wrong had the best model fit.

For 29 out of 45 countries, the proposed Mislevy-Wu model outperformed the suggested model with a latent response propensity in terms of a GHP difference of at least 0.001. Overall, these findings indicated that the models assuming ignorability or latent ignorability performed worse in terms of model fit compared to scaling models that acknowledge the dependence of responding to an item from the true but occasionally unobserved item response.

### 5.3. Country-Specific Model Parameters for Latent Ignorable Model and Mislevy-Wu Model

Now, we present findings of model parameters characterizing the missingness mechanism from the model MO2 relying on latent ignorability and the Mislevy-Wu model MM2. The parameters are shown in Table 6. The SD of the latent response propensity SD(ξ) was somewhat lower in the Mislevy-Wu model (MM2, with a median Med=1.98) than the model assuming latent ignorability (MO2, Med=1.93). Moreover, by additionally including the latent item response as a predictor for the response indicator, the correlation Cor(θ,ξ) between the latent ability θ and response propensity ξ was slightly lower in model MM2 (Med=0.43) than MO2 (Med=0.46). Most importantly, the missingness mechanism strongly differed between CR and MC items. The median δ parameter in model MM2 for CR items was −2.61, indicating that students that did not know the item had a higher probability of omitting the item even after controlling for the latent response propensity ξ. In contrast, the median δ parameter was −0.48. Hence, there was a smaller influence of (latently) knowing the item with the response indicators. However, it was different from zero for most countries, indicating that the model MO2 assuming latent ignorability did not adequately explain the missingness mechanism. Overall, it can be seen that those model parameters strongly vary across countries. Hence, it can be concluded that assuming different missingness mechanisms for countries could have non-negligible consequences for country rankings (see [126]).

### 5.4. Country Means and Country Standard Deviations Obtained from Different Scaling Models

For comparing country means, 11 out of 19 specified scaling models were selected to contrast the dissimilarity of country mean and standard deviation estimates. Based on the findings of the similarity of models in Section 5.1 (see Table 4), 8 out of 19 models were omitted in the reporting of the comparisons because they provided very similar findings to at least one of the 11 reported models. Table 7 shows the country means of these 11 different treatments of missing item responses. The country rank (column “${\mathrm{rk}}_{\mathrm{UW}}$”) serves as the reference for the comparison among methods. Moreover, the interval of country ranks obtained from the different methods are shown in column “${\mathrm{rk}}_{\mathrm{Int}}$”. The average maximum difference in country ranks was 2.4 (SD=1.8) and ranged between 0 (SGP, HKG, EST, DEU, LUX, BIH) and 8 (IRL). The range in country means (i.e., the difference of the largest and smallest country mean of the 11 methods) was noticeable (M=5.0) and showed strong variability between countries (SD=2.8, Min=1.5, Max=12.5). Interestingly, large range values were obtained for countries with missing proportions that were strongly below and above the average missing proportion. For example, Ireland (IRL) had a relatively low missing rate of 5.8% and reached rank 15 with method UW (M=505.2) that treated missing item responses as wrong. Methods that ignore missing item responses resulted in a lower country mean (UO1: M=499.9; MO2: M=500.7; IO2: M=500.0). In contrast, the Mislevy-Wu model (MM2 and IO2)—which also takes the relation of the response indicator and the true item response into account—resulted in higher country means (MM2: M=505.1; IO2: M=504.9). Across the 11 estimation methods, Ireland reached ranks between 15 and 23 which can be considered a large variability. Moreover, the range of country means for Ireland was 8.2, which is two to three times higher than standard errors for country means due to the sampling of students in PISA. Italy (ITA, rank 26; M=492.0) that had a relatively high missing rate of 12.4% profit by ignoring missing item responses assuming latent ignorability (UO1: M=494.7; MO2: M=494.4; IO2: M=494.0). However, the Mislevy-Wu model produced considerably lower scores (MO2: M=490.1; IO2: M=489.9). An interesting case is Sweden (SWE, rank 25) that had a high missing proportion rate of 12.7%, but almost half of missing item responses (i.e., 5.9%) stemmed from not reached responses. This not reached proportion was the highest among all countries in our study. Sweden had rank 25 when treating missing item responses as wrong (UW: M=491.8), but strongly profits in models that ignore the not reached items (UN1: M=499.1) or treated the proportion of not reached items as a predictor in the latent background model (UN2: M=499.7). If also omitted items would be treated as (latent) ignorable, the country mean for Sweden further increased (UO1: M=501.3; MO2: M=501.1; IO2: M=501.3). In contrast to many other countries, the country means obtained from the Mislevy-Wu model (MM2: M=497.9; IO2: M=498.0) were also much larger than the country mean obtained by treating missing items as wrong (UW: M=491.8).

In Table A3 in Appendix C, standard errors for country means are shown. Across different models and countries, the average standard error was 2.20 (SD = 0.47, Min = 1.21, Max = 3.65). Within a country, the variability (i.e., standard deviation (column SD) in Table A3) of standard errors for the mean was small (M = 0.05, SD = 0.05, Min = 0.01, Max = 0.21).

In Table A4 in Appendix C, standard errors for differences in means stemming from two different models are displayed. We consider differences between the models UW, MO2 and MM2. It turned out that the standard error for mean differences between two models was very small compared to the standard error for the mean for a single model. The largest average standard errors were obtained for the mean difference between models UW2 and MO2 (see column “UW-MO2” in Table A4; M = 0.037, SD = 0.036, Min = 0, Max = 0.149). These two models represent the two extreme missing data treatments that explain the observation of obtaining the largest standard errors. The smallest standard errors were obtained for the model difference between UW and MM2 (column “UW-MM2”; M = 0.021, SD = 0.019, Min = 0.000, Max = 0.096). The average standard errors for the mean difference between the models MO2 and MM2 was 0.027 (column “UW-MO2”; M = 0.022, SD = 0.019, Min = 0.001, Max = 0.093).

The estimates of country standard deviations stemming from different models for the missing data treatment are shown in Table A5 in Appendix C. As in the case of the country mean, it turned out that model choice also impacted standard deviations. Within a country, the standard deviation of the different standard deviation estimates showed nonnegligible variability (column “SD” in Table A5; M = 1.25, SD = 0.96, Min = 0.3, Max = 5.4). The within-country ranges of country standard deviations across models were even larger than for country means.

## 6. Discussion

In this paper, competing approaches for handling missing item responses in educational large-scale assessment studies like PISA are investigated. We compared the Mislevy-Wu model that allows the probability of item missingness depending on the item itself with the more frequently discussed approaches of scoring items as wrong or models assuming latent ignorability. In an illustrative simulation study, we demonstrated that neither of the two latter approaches provides unbiased parameter estimates if the more general Mislevy-Wu model holds (see also [44]). In realistic data constellations in which the Mislevy-Wu model holds, it is likely that the method of scoring missing item responses as wrong results in underestimated (country) means, while models relying on latent ignorability provide overestimated means. Based on these findings, we are convinced that the often-taken view in psychometric literature that strongly advocates latent ignorability and denies the scoring as wrong [4,11,12,18] is unjustified (see also [24,25,27]).

In our reanalysis of the PISA 2018 mathematics data, different scaling models with different treatments of missing item responses were specified. It has been shown that differences in country means and country standard deviations across models can be substantial. The present study sheds some light on the ongoing debate about properly handling missing item responses in educational large-scale assessment studies. Ignoring missing item responses and treating them as wrong can be seen as opposing strategies. Other scaling models can be interpreted to provide results somewhere between these two extreme poles of handling missingness. We argued that the Mislevy-Wu model contains the strategy of scoring as wrong and the latent ignorable model as submodels. Hence, these missing data treatments can be tested. In our analysis, it turned out that the Mislevy-Wu model fitted the PISA data best. More importantly, the treatment of missing item responses as wrong provided a better model fit than ignoring them or modeling them by the latent ignorable model that has been strongly advocated in the past [10,11]. It also turned out that the missingness mechanism strongly differed between CR and MC items.

We believe that the call for controlling for test-taking behavior in the reporting in large-scale assessment studies such as response propensity [4] using models that also include response times [157,158] poses a threat to validity [159,160,161,162,163,164] because results can be simply manipulated by instructing students to omit items they do not know [26]. Notably, missing item responses are mostly omissions for CR items. Response times might be useful for detecting rapid guessing or noneffortful responses [81,165,166,167,168,169,170,171]. However, it seems likely that students who do not know the solution to CR items do not respond to these items. In this case, the latent ignorability assumption is unlikely to hold, and scaling models that rely on it (see [4,12]) will result in biased and unfair country comparisons. We are skeptical that the decision of whether a missing item response is scored as wrong should be based on a particular response time threshold [166,172,173]. Students can also be simply instructed to quickly skip items that they are not probably able to solve.

In our PISA analysis, we restricted the analysis to 45 countries that received booklets of average item difficulty. Recently, a number of low-performing countries also participated in recent PISA cycles that receive booklets of lower difficulty [174,175,176]. We did not include these low-performing countries for the following reasons. First, the proportion of correctly solved items for low-performing countries is lower. This implies that it is more difficult for these countries to disentangle the parameters of the model for response indicators and item parameters. Second, the meaning of missingness on item responses across countries differs if different booklets are administered in countries. Hence, it is difficult to compare outcomes of different scaling models for the missing data treatment if there is no prerequisite of the same administered test design. To some extent, the issue also appears in the recently implemented multi-stage testing (MST; [177,178]) design in PISA that also results in different proportions of test booklets of different average difficulty across countries. We think that there is no defensible strategy of properly treating missing item responses from MST designs that enables a fair and valid comparison of countries [26].

In this article, we only investigated the impact of missing item responses on country means and country standard deviations. In LSA studies, missing data is also a prevalent issue for student covariates (e.g., sociodemographic status; see [104,179,180,181,182,183,184]). As covariates also enter the plausible value imputation of latent abilities through the latent background model [75,129] or relationships of abilities and covariates are often of interest in reporting, missing data on covariates is also a crucial issue that needs to be adequately addressed [104].

It could be argued that there is not a unique, scientifically sound, or widely publicly accepted scaling model in PISA (see [185]). The uncertainty in choosing a psychometric model can be reflected by explicitly acknowledging the variability of country means and standard deviations obtained by different model assumptions. This additional source of variance associated with model uncertainty [186,187,188,189,190,191] can be added to the standard error due to the sampling of students and linking error due to the selection of items [192]. The assessment of specification uncertainty has been discussed in sensitivity analysis [193] and has recently become popular as multiverse analysis [194,195] or specification curve analysis [196,197]. As educational LSA studies are policy-relevant [198,199], we think that model uncertainty should be included in statistical inference [200,201].

## Figures and Tables

**Table 1:** Bias for the mean and the standard deviation for different missing data treatments as a function of the missing proportion and the missingness parameter δ.

δ	Mean					Standard Deviation				
	−10	−3	−2	−1	0	−10	−3	−2	−1	0
Model										
	*5% missing data*									
CD	−0.002	−0.006	−0.006	−0.007	−0.004	−0.005	−0.008	−0.009	−0.010	−0.007
MM1	−0.005	−0.005	−0.006	−0.008	−0.007	−0.007	−0.007	−0.009	−0.010	−0.010
UW	−0.002	−0.005	−0.022	**−0.041**	**−0.065**	−0.005	−0.004	−0.003	−0.006	−0.005
UO	**−0.090**	**−0.084**	**−0.081**	**−0.058**	−0.021	**−0.040**	**−0.036**	**−0.039**	−0.027	−0.015
MO2	**−0.085**	**−0.077**	**−0.071**	**−0.044**	−0.005	**−0.037**	**−0.032**	**−0.033**	−0.021	−0.009
IF1	**−0.090**	**−0.086**	**−0.082**	**−0.058**	−0.022	**−0.039**	**−0.036**	**−0.039**	−0.026	−0.014
IF2	**−0.088**	**−0.082**	**−0.078**	**−0.052**	−0.008	**−0.037**	**−0.034**	**−0.037**	−0.025	−0.009
	*10% missing data*									
CD	−0.005	−0.006	−0.005	−0.005	−0.008	−0.009	−0.009	−0.008	−0.008	−0.011
MM1	−0.009	−0.008	−0.009	−0.006	−0.002	−0.011	−0.010	−0.012	−0.008	−0.007
UW	−0.005	−0.022	**−0.049**	**−0.083**	**−0.139**	−0.009	−0.000	−0.004	−0.005	−0.015
UO	**−0.159**	**−0.136**	**−0.113**	**−0.090**	**−0.037**	**−0.090**	**−0.064**	**−0.047**	**−0.039**	−0.023
MO2	**−0.149**	**−0.123**	**−0.103**	**−0.075**	−0.006	**−0.079**	**−0.052**	**−0.040**	**−0.035**	−0.010
IF1	**−0.160**	**−0.139**	**−0.116**	**−0.092**	**−0.038**	**−0.089**	**−0.065**	**−0.047**	**−0.040**	−0.022
IF2	**−0.152**	**−0.132**	**−0.109**	**−0.083**	−0.012	**−0.080**	**−0.057**	**−0.042**	**−0.038**	−0.014
	*20% missing data*									
CD	−0.004	−0.005	−0.002	−0.004	−0.004	−0.007	−0.009	−0.005	−0.008	−0.006
MM1	−0.018	−0.005	−0.005	−0.008	−0.007	−0.017	−0.009	−0.009	−0.012	−0.011
UW	−0.004	**−0.072**	**−0.129**	**−0.198**	**−0.268**	−0.006	−0.005	−0.014	−0.019	−0.022
UO	**−0.203**	**−0.211**	**−0.183**	**−0.144**	**−0.064**	**−0.148**	**−0.129**	**−0.095**	**−0.073**	**−0.038**
MO2	**−0.203**	**−0.210**	**−0.175**	**−0.115**	−0.005	**−0.146**	**−0.126**	**−0.088**	**−0.053**	−0.007
IF1	**−0.208**	**−0.214**	**−0.183**	**−0.148**	**−0.063**	**−0.147**	**−0.129**	**−0.091**	**−0.073**	**−0.033**
IF2	**−0.212**	**−0.211**	**−0.183**	**−0.126**	−0.010	**−0.152**	**−0.121**	**−0.089**	**−0.059**	−0.011
	*30% missing data*									
CD	−0.008	−0.006	−0.004	−0.004	−0.006	−0.010	−0.008	−0.008	−0.009	−0.011
MM1	**−0.054**	−0.008	−0.008	−0.010	−0.005	**−0.122**	−0.012	−0.011	−0.013	−0.005
UW	−0.006	**−0.159**	**−0.225**	**−0.298**	**−0.363**	−0.009	−0.021	−0.018	−0.014	−0.002
UO	**−0.198**	**−0.238**	**−0.226**	**−0.179**	**−0.070**	**−0.211**	**−0.165**	**−0.132**	**−0.094**	**−0.042**
MO2	**−0.192**	**−0.239**	**−0.228**	**−0.159**	−0.001	**−0.213**	**−0.165**	**−0.133**	**−0.083**	−0.008
IF1	**−0.208**	**−0.244**	**−0.231**	**−0.183**	**−0.074**	**−0.210**	**−0.165**	**−0.134**	**−0.092**	**−0.039**
IF2	**−0.202**	**−0.247**	**−0.233**	**−0.168**	−0.010	**−0.211**	**−0.166**	**−0.130**	**−0.086**	−0.013

*Note.* CD = complete-data analysis; UW = scoring as wrong (Section 2.1); MM1 = Mislevy-Wu model with common d parameter (Section 2.5, Equation (14)); UO = ignoring missing item responses (Section 2.3); MO2 = model-based latent ignorability (Section 2.4, Equations (10) and (11); IF1 = FCS imputation based on item responses (Section 2.6); IF2 = FCS imputation based on item responses and response indicators (Section 2.6); Absolute biases values larger than 0.03 are printed in bold.

**Table 2:** Descriptive statistics of the PISA 2018 mathematics sample.

Country	*N*	*I*	$N_{\mathrm{item}}$ 1	$M_{\mathrm{OECD}}$	${\mathrm{SD}}_{\mathrm{OECD}}$	$M_{\mathrm{stand}}$	%NA	%NR	%${\mathrm{NA}}_{\mathrm{CR}}$	%${\mathrm{NA}}_{\mathrm{MC}}$
ALB	2609	69	787.0	438.0	83.4	446.0	8.0	1.9	11.4	2.6
AUS	7705	70	2367.1	491.7	92.9	501.8	7.3	2.4	10.3	2.5
AUT	3731	70	1133.7	499.1	92.7	509.6	8.4	1.8	12.5	2.0
BEL	4696	70	1393.0	507.8	95.6	518.6	8.3	2.6	11.9	2.5
BIH	3512	70	1071.0	406.5	82.0	413.1	**18.8** 1	**3.9**	27.9	4.2
BLR	3141	70	967.8	470.7	92.4	480.0	7.8	2.4	11.4	2.1
BRN	2812	69	845.0	430.6	91.3	438.2	6.1	1.7	8.8	1.8
CAN	9782	70	2786.3	511.7	92.4	522.7	5.8	2.2	8.2	2.1
CHE	3141	70	964.5	514.5	93.4	525.6	8.2	2.5	11.9	2.2
CZE	3798	70	1164.0	498.5	93.4	509.0	9.2	2.0	13.9	1.9
DEU	3000	70	908.6	499.0	95.9	509.5	9.6	2.5	14.0	2.4
DNK	4354	70	1250.1	510.7	81.3	521.7	5.9	2.0	8.6	1.7
ESP	14,7681	70	4408.3	481.7	88.3	491.5	**10.6** 1	2.9	15.5	2.7
EST	2880	70	890.9	523.8	81.6	535.3	6.6	2.0	9.6	1.8
FIN	3056	70	935.0	505.7	83.3	516.4	8.9	3.0	12.8	2.7
FRA	3405	70	1046.8	495.5	92.2	505.8	**10.1** 1	3.0	14.8	2.6
GBR	7063	70	2174.0	502.1	92.9	512.7	8.2	2.5	11.8	2.5
GRC	2634	70	790.4	451.1	89.5	459.6	**10.7** 1	2.7	15.7	2.6
HKG	2484	70	748.0	551.0	92.5	563.5	**3.9**	0.8	5.8	0.8
HRV	2683	70	805.2	464.5	87.1	473.6	**11.8** 1	2.7	17.6	2.5
HUN	2785	70	857.4	482.3	91.2	492.0	8.6	2.0	13.0	1.7
IRL	3031	70	935.5	500.2	78.1	510.7	5.8	1.3	8.7	1.2
ISL	1807	70	545.1	493.8	90.8	504.1	9.7	**4.4**	12.9	4.5
ISR	2825	70	846.6	464.0	107.5	473.0	**12.1** 1	**4.5**	16.9	4.5
ITA	6401	70	1978.9	485.9	94.0	495.8	**12.4** 1	2.8	18.9	2.1
JPN	3302	70	1018.6	527.4	87.1	539.1	8.4	1.9	12.9	1.4
KOR	2741	70	823.1	525.9	100.4	537.5	6.4	1.7	9.4	1.6
LTU	2824	70	846.3	480.1	90.0	489.8	7.4	1.5	11.4	1.1
LUX	2827	70	872.0	481.3	98.6	491.0	**10.4** 1	2.8	15.3	2.7
LVA	2190	70	656.4	498.5	80.5	509.0	6.4	1.7	9.7	1.1
MLT	1383	69	415.8	469.5	101.6	478.8	9.8	**3.9**	13.5	3.6
MNE	3595	70	1109.7	430.8	83.0	438.4	**17.3** 1	**3.8**	25.9	3.5
MYS	3284	70	1000.8	440.2	82.0	448.2	**1.2**	0.6	1.5	0.7
NLD	2939	70	742.6	518.4	92.9	529.7	**4.4**	1.1	6.7	0.9
NOR	3141	70	969.5	502.4	90.3	513.0	**10.7** 1	**3.7**	15.1	3.7
NZL	3309	70	1021.2	495.6	93.0	506.0	8.1	2.2	11.7	2.3
POL	3022	70	932.6	515.8	90.5	526.9	7.1	1.9	10.7	1.3
PRT	3202	70	987.6	493.1	96.2	503.3	**10.6** 1	2.8	15.8	2.3
RUS	3131	70	939.3	487.8	87.4	497.8	7.9	2.2	11.6	2.1
SGP	2732	70	822.3	570.3	93.3	583.6	**2.7**	0.8	3.8	0.8
SVK	2514	70	727.9	484.6	100.1	494.5	8.0	1.8	11.9	1.7
SVN	3519	70	1054.7	509.5	88.7	520.4	7.1	1.5	10.7	1.4
SWE	2982	70	918.7	502.8	90.3	513.4	**12.7** 1	**5.9**	17.3	5.4
TUR	3723	70	1147.8	453.4	87.4	462.0	6.7	1.6	9.7	1.8
USA	2629	70	804.9	478.0	92.4	487.6	**4.0**	2.0	5.2	1.9

*Note.N* = number of students; *I* = number of items; $N_{\mathrm{item}}$ = average number of students per item; $N_{\mathrm{OECD}}$ = officially reported country mean by OECD [16]; $M_{\mathrm{OECD}}$ = officially reported country standard deviation by OECD [16]; Mstand = standardized country mean (M = 500 and SD = 100 in total population); %NA = proportion of item responses with missing data; %NR = proportion of item responses that are not reached; %${\mathrm{NA}}_{\mathrm{CR}}$ = proportion of constructed-response item responses with missing data; %${\mathrm{NA}}_{\mathrm{MC}}$ = proportion of multiple-choice item responses with missing data; Missing item response rates larger than 10.0% and smaller than 5.0% are printed in bold. Missing rates for not reached responses larger than 3.0% are printed in bold. See Appendix B for country labels.

**Table 3:** Overview of 19 specified scaling models for the treatment of missing item responses in the PISA 2018 mathematics case study.

Model	Ref.	Description
UW	Section 2.1	response indicators unmodeled: scoring as wrong
MW	Section 2.5	model-based treatment: treatment as wrong in the Mislevy-Wu model by setting $\rho_{i}=-10$ in Equation (14)
IW	Section 2.5	imputation-based treatment on the IRT model MW: imputation as wrong based on the Mislevy-Wu model and setting $\rho_{i}=-10$ in Equation (14)
UP	Section 2.2	response indicators unmodeled: multiple-choice items scored as partially correct
IP	Section 2.2	imputation-based treatment: multiple-choice items imputed with probabilities $1/K_{i}$, for correct response where $K_{i}$ is the number of response alternatives
UN1	Section 4.2.1	response indicators unmodeled: not reached items ignored in the scaling model
UN2	Section 4.2.1	response indicators unmodeled: proportion of not reached items included as a predictor in the latent background model
UO1	Section 2.3	response indicators unmodeled: missing item responses ignored in the scaling model
MO1	Section 2.4	model-based treatment: model-based ignorability specified as the Mislevy-Wu model with $\delta_{i}=0$ and Cor(θ,ξ)=0
IO1	Section 2.4.1	imputation-based treatment on the IRT model MO1
UO1	Section 2.3 and Section 4.2.1	response indicators unmodeled: including proportion of missing item responses in the latent background model
MO2	Section 2.4	model-based treatment: model-based latent ignorability specified as the Mislevy-Wu model with $\delta_{i}=0$
IO2	Section 2.4.1	imputation-based treatment on the IRT model MO2
MM1	Section 2.5	model-based treatment: Mislevy-Wu model with common $\delta_{i}$ parameter
IM1	Section 2.4.1	imputation-based treatment on the IRT model MM1
MM2	Section 2.5	model-based treatment: Mislevy-Wu model with item-format specific $\delta_{i}$ parameter
IM2	Section 2.4.1	imputation-based treatment on the IRT model MM2
IF1	Section 2.6 and Section 4.2.2	imputation-based treatment on fully conditional specification: using predictive mean matching for item responses $X_{p}$ separately for each test booklet
IF2	Section 2.6 and Section 4.2.2	imputation-based treatment on fully conditional specification: using predictive mean matching for item responses $X_{p}$ and response indicators $R_{p}$ separately for each test booklet

*Note.* Ref. = reference in this article.

**Table 4:** Average absolute differences in country means of different treatments of missing item responses.

	UW	MW	IW	UP	IP	UN1	UN2	UO1	MO1	IO1	MO2	UO2	IO2	MM1	IM1	MM2	IM2	IF1	IF2
UW	—	**0.3**	**0.0**	**0.7**	**0.8**	1.9	1.7	3.0	3.0	3.0	2.6	2.8	2.6	1.4	1.5	1.6	1.5	3.0	2.8
MW	**0.3**	—	**0.3**	**0.9**	**0.9**	2.0	1.7	3.0	3.0	3.0	2.7	2.8	2.6	1.4	1.6	1.6	1.6	3.1	2.8
IW	**0.0**	**0.3**	—	**0.7**	**0.8**	1.9	1.7	3.0	3.0	3.0	2.6	2.8	2.6	1.4	1.5	1.6	1.5	3.0	2.8
UP	**0.7**	**0.9**	**0.7**	—	**0.3**	1.4	1.5	2.7	2.7	2.7	2.4	2.4	2.3	1.1	1.2	1.3	1.2	2.7	2.6
IP	**0.8**	**0.9**	**0.8**	**0.3**	—	1.5	1.5	2.7	2.7	2.7	2.4	2.4	2.3	1.2	1.2	1.4	1.3	2.7	2.6
UN1	1.9	2.0	1.9	1.4	1.5	—	**1.0**	2.1	2.1	2.1	2.0	1.8	1.9	**1.0**	**1.0**	**0.9**	**0.9**	2.2	2.6
UN2	1.7	1.7	1.7	1.5	1.5	**1.0**	—	2.4	2.5	2.5	2.0	2.2	2.0	1.4	1.4	1.2	1.3	2.6	2.7
UO1	3.0	3.0	3.0	2.7	2.7	2.1	2.4	—	**0.0**	**0.2**	**0.7**	**0.3**	**0.6**	2.5	2.5	2.2	2.3	**0.7**	1.9
MO1	3.0	3.0	3.0	2.7	2.7	2.1	2.5	**0.0**	—	**0.2**	**0.7**	**0.4**	**0.7**	2.5	2.5	2.2	2.3	**0.7**	1.9
IO1	3.0	3.0	3.0	2.7	2.7	2.1	2.5	**0.2**	**0.2**	—	**0.7**	**0.4**	**0.7**	2.6	2.5	2.3	2.4	**0.8**	1.9
MO2	2.6	2.7	2.6	2.4	2.4	2.0	2.0	**0.7**	**0.7**	**0.7**	—	**0.6**	**0.4**	2.2	2.3	1.8	2.0	**1.0**	1.8
UO2	2.8	2.8	2.8	2.4	2.4	1.8	2.2	**0.3**	**0.4**	**0.4**	**0.6**	—	**0.5**	2.3	2.2	2.0	2.1	**0.8**	1.8
IO2	2.6	2.6	2.6	2.3	2.3	1.9	2.0	**0.6**	**0.7**	**0.7**	**0.4**	**0.5**	—	2.2	2.2	1.8	2.0	**1.0**	1.8
MM1	1.4	1.4	1.4	1.1	1.2	**1.0**	1.4	2.5	2.5	2.6	2.2	2.3	2.2	—	**0.4**	**0.6**	**0.5**	2.6	2.7
IM1	1.5	1.6	1.5	1.2	1.2	**1.0**	1.4	2.5	2.5	2.5	2.3	2.2	2.2	**0.4**	—	**0.8**	**0.7**	2.6	2.6
MM2	1.6	1.6	1.6	1.3	1.4	**0.9**	1.2	2.2	2.2	2.3	1.8	2.0	1.8	**0.6**	**0.8**	—	**0.4**	2.3	2.5
IM2	1.5	1.6	1.5	1.2	1.3	**0.9**	1.3	2.3	2.3	2.4	2.0	2.1	2.0	**0.5**	**0.7**	**0.4**	—	2.4	2.5
IF1	3.0	3.1	3.0	2.7	2.7	2.2	2.6	**0.7**	**0.7**	**0.8**	**1.0**	**0.8**	**1.0**	2.6	2.6	2.3	2.4	—	1.9
IF2	2.8	2.8	2.8	2.6	2.6	2.6	2.7	1.9	1.9	1.9	1.8	1.8	1.8	2.7	2.6	2.5	2.5	1.9	—

*Note.* Mean absolute differences smaller or equal than 1.0 are printed in bold.

**Table 5:** Model comparisons based on the Bayesian information crierion (BIC) and the Gilula–Haberman penalty (GHP).

Country	BIC					GHP					
	MW	MO1	MO2	MM1	MM2	MW	MO1	MO2	MM1	MM2	Diff
ALB	63663	63754	63600	**63579**	63586	0.6423	0.6433	0.6416	0.6414	0.6414	0.0003
AUS	193304	194008	193316	193145	**193105**	0.6321	0.6344	0.6321	0.6315	0.6314	0.0007
AUT	97019	97685	97174	97007	**96993**	0.6618	0.6664	0.6628	0.6616	0.6615	**0.0013**
BEL	118264	119131	118426	118236	**118186**	0.6665	0.6715	0.6675	0.6664	0.6660	**0.0014**
BIH	98447	98779	98534	98371	**98359**	0.7101	0.7125	0.7107	0.7095	0.7093	**0.0014**
BLR	82460	82729	82564	82455	**82396**	0.6509	0.6531	0.6517	0.6508	0.6503	**0.0014**
BRN	62751	62864	62756	**62715**	62719	0.5925	0.5936	0.5925	0.5921	0.5921	0.0005
CAN	213551	214215	213549	213382	**213268**	0.6316	0.6336	0.6316	0.6311	0.6307	0.0009
CHE	84792	85329	84940	84777	**84743**	0.6724	0.6768	0.6736	0.6722	0.6719	**0.0017**
CZE	102441	102838	102508	102382	**102301**	0.6780	0.6807	0.6784	0.6776	0.6770	**0.0015**
DEU	79134	79714	79219	79118	**79102**	0.6729	0.6779	0.6736	0.6727	0.6725	**0.0011**
DNK	97368	97632	97328	**97270**	97277	0.6232	0.6249	0.6229	0.6225	0.6225	0.0004
ESP	377203	378998	377528	377027	**376832**	0.6844	0.6877	0.6850	0.6841	0.6837	**0.0013**
EST	74697	74921	74716	74639	**74623**	0.6384	0.6404	0.6386	0.6379	0.6377	0.0009
FIN	80421	80504	80386	80315	**80228**	0.6602	0.6609	0.6599	0.6592	0.6585	**0.0014**
FRA	92877	93593	93019	92868	**92833**	0.6820	0.6874	0.6830	0.6819	0.6816	**0.0015**
GBR	181680	182770	181704	181518	**181471**	0.6457	0.6496	0.6458	0.6451	0.6449	0.0009
GRC	68339	68606	68485	68317	**68269**	0.6814	0.6841	0.6829	0.6811	0.6805	**0.0023**
HKG	57050	57459	57113	57054	**57048**	0.5965	0.6009	0.5972	0.5965	0.5964	0.0008
HRV	70685	71044	70791	70679	**70669**	0.6927	0.6963	0.6937	0.6926	0.6924	**0.0013**
HUN	72125	72492	72187	72080	**72060**	0.6437	0.6470	0.6442	0.6432	0.6430	**0.0013**
IRL	77409	77712	77432	77381	**77369**	0.6323	0.6349	0.6325	0.6320	0.6319	0.0006
ISL	48098	48071	48043	48006	**47965**	0.6782	0.6779	0.6774	0.6768	0.6761	**0.0013**
ISR	62551	62964	62675	62531	**62520**	0.6771	0.6817	0.6785	0.6768	0.6766	**0.0018**
ITA	179041	180275	179253	178956	**178914**	0.6951	0.6999	0.6959	0.6947	0.6945	**0.0014**
JPN	87938	88375	87998	87917	**87858**	0.6606	0.6639	0.6610	0.6604	0.6599	**0.0012**
KOR	65114	65613	65110	65067	**65066**	0.6229	0.6278	0.6229	0.6224	0.6223	0.0005
LTU	68816	69098	68893	68797	**68788**	0.6411	0.6439	0.6419	0.6409	0.6408	**0.0011**
LUX	79066	79552	79236	79051	**79033**	0.6933	0.6976	0.6948	0.6931	0.6929	**0.0019**
LVA	53764	53922	53754	53731	**53728**	0.6441	0.6461	0.6439	0.6436	0.6435	0.0005
MLT	33418	33625	33404	**33370**	33371	0.6325	0.6367	0.6323	0.6315	0.6314	0.0008
MNE	103907	104412	104044	103857	**103833**	0.7174	0.7210	0.7183	0.7170	0.7168	**0.0016**
MYS	**66244**	66271	66256	66246	66253	0.5042	0.5045	0.5043	0.5042	0.5042	0.0001
NLD	50077	50286	50125	50063	**50055**	0.5869	0.5895	0.5875	0.5867	0.5865	**0.0010**
NOR	86955	87260	87005	86842	**86802**	0.6859	0.6884	0.6863	0.6850	0.6846	**0.0017**
NZL	87003	87519	87077	86965	**86951**	0.6514	0.6554	0.6520	0.6511	0.6509	**0.0010**
POL	78675	78987	78675	78616	**78599**	0.6441	0.6468	0.6441	0.6436	0.6434	0.0007
PRT	89473	89900	89627	89457	**89322**	0.6933	0.6967	0.6945	0.6931	0.6920	**0.0025**
RUS	78318	78563	78384	78290	**78262**	0.6588	0.6610	0.6594	0.6586	0.6583	**0.0011**
SGP	58480	58724	58515	**58466**	58466	0.5576	0.5600	0.5579	0.5574	0.5573	0.0006
SVK	59699	59958	59788	59692	**59671**	0.6593	0.6622	0.6602	0.6591	0.6588	**0.0014**
SVN	88287	88818	88451	88292	**88245**	0.6518	0.6558	0.6530	0.6518	0.6514	**0.0016**
SWE	86292	86416	86272	86145	**86037**	0.7188	0.7199	0.7187	0.7175	0.7166	**0.0021**
TUR	96064	96326	96230	96041	**96032**	0.6412	0.6430	0.6423	0.6410	0.6409	**0.0014**
USA	61234	61223	61167	61154	**61147**	0.5806	0.5806	0.5800	0.5798	0.5797	0.0003

*Note.* BIC values for best-performing model printed in bold. GHP differences (column Diff) between models MO2 and MM2 larger than 0.001 printed in bold. See Appendix B for country labels.

**Table 6:** Model parameters from the latent ignorable model (MO2) and the Mislevy-Wu Model (MM2).

Country	MO2		MM2			
	SD(ξ)	Cor(θ,ξ)	SD(ξ)	Cor(θ,ξ)	− $\delta_{\mathrm{CR}}$	− $\delta_{\mathrm{MC}}$
ALB	2.50	0.42	2.47	0.44	−1.23	−0.91
AUS	2.59	0.46	2.52	0.46	−2.31	−0.71
AUT	1.90	0.54	1.79	0.49	−3.42	−1.01
BEL	1.92	0.56	1.83	0.51	−3.10	−0.43
BIH	1.87	0.40	1.82	0.43	−2.12	−0.53
BLR	1.81	0.35	1.79	0.29	−2.95	0.43
BRN	2.21	0.33	2.17	0.33	−2.08	−1.08
CAN	2.30	0.44	2.26	0.41	−2.37	−0.09
CHE	1.91	0.50	1.83	0.44	−3.12	−0.46
CZE	1.73	0.43	1.68	0.35	−2.46	0.46
DEU	1.91	0.57	1.80	0.53	−2.63	−0.48
DNK	2.25	0.43	2.19	0.43	−1.73	−1.32
ESP	1.83	0.47	1.77	0.45	−2.45	−0.01
EST	2.10	0.41	2.06	0.36	−2.43	−0.35
FIN	1.99	0.31	2.00	0.28	−2.22	0.57
FRA	1.85	0.57	1.74	0.52	−3.19	−0.52
GBR	2.48	0.57	2.38	0.56	−2.26	−0.41
GRC	1.80	0.33	1.78	0.30	−3.59	−0.24
HKG	2.34	0.60	2.22	0.52	−4.07	−0.67
HRV	1.89	0.46	1.83	0.45	−2.99	−0.64
HUN	2.17	0.49	2.11	0.45	−2.48	−0.15
IRL	1.97	0.47	1.91	0.44	−2.23	−0.01
ISL	2.35	0.22	2.36	0.23	−2.00	0.06
ISR	2.36	0.50	2.26	0.49	−3.04	−1.28
ITA	1.75	0.54	1.65	0.49	−2.69	−0.49
JPN	1.92	0.49	1.84	0.43	−2.67	0.45
KOR	2.61	0.64	2.49	0.62	−2.15	−0.80
LTU	1.89	0.42	1.84	0.36	−3.20	−0.69
LUX	1.76	0.47	1.68	0.41	−3.01	−0.73
LVA	1.98	0.44	1.93	0.41	−1.86	−0.08
MLT	2.94	0.61	2.86	0.62	−2.03	−0.82
MNE	1.86	0.47	1.81	0.49	−2.61	−0.57
MYS	2.42	0.18	2.43	0.15	−1.94	−2.76
NLD	2.37	0.45	2.32	0.40	−3.07	−0.61
NOR	2.11	0.42	2.05	0.41	−2.64	−0.64
NZL	2.19	0.53	2.09	0.50	−2.56	−0.60
POL	2.05	0.48	1.99	0.42	−2.12	−0.08
PRT	1.76	0.42	1.72	0.34	−2.72	1.20
RUS	2.00	0.38	1.97	0.35	−2.79	−0.28
SGP	2.51	0.50	2.43	0.44	−2.80	−1.11
SVK	1.93	0.41	1.88	0.36	−3.15	−0.23
SVN	1.85	0.49	1.77	0.42	−9.99	−0.34
SWE	1.90	0.32	1.89	0.30	−2.24	0.01
TUR	1.71	0.26	1.68	0.18	−4.07	−1.40
USA	2.72	0.26	2.70	0.26	−1.54	−0.28

*Note.* standard deviation of latent propensity variable ξ; Cor(θ,ξ) = correlation of latent ability θ with latent propensity variable ξ; $\delta_{\mathrm{CR}}$ = common δ parameter for constructed response items; $\delta_{\mathrm{MC}}$ = common δ parameter for multiple-choice items. See Appendix B for country labels.

**Table 7:** Country means for PISA 2018 mathematics from 11 different scaling models for missing item responses.

Country	%NA	%NR	${\mathrm{rk}}_{\mathrm{UW}}$	${\mathrm{rk}}_{\mathrm{Int}}$	Aver	SD	rg	UW	UP	UN1	UN2	UO1	MO2	IO2	MM2	IM2	IF1	IF2
SGP	**2.7**	0.8	1	1–1	568.1	1.5	**5.3**	568.0	567.8	567.6	567.4	567.7	567.0	567.7	567.3	567.8	568.7	572.4
HKG	**3.9**	0.8	2	2–2	548.9	1.3	4.1	550.1	550.0	548.3	548.3	548.2	548.0	547.9	548.3	548.4	548.8	552.0
NLD	**4.4**	1.1	3	3–4	531.4	0.6	2.1	531.6	531.5	531.7	531.6	530.9	530.7	531.1	531.2	531.5	530.9	532.9
JPN	8.4	1.9	4	3–4	532.1	1.8	4.6	530.8	530.6	530.0	530.3	533.8	533.9	533.9	531.1	531.0	533.5	534.6
EST	6.6	2.0	5	5–5	526.7	1.0	3.4	527.9	529.2	526.5	526.8	525.7	526.2	526.4	526.8	525.9	526.1	526.1
KOR	6.4	1.7	6	6–7	522.5	1.3	4.4	523.7	523.6	523.1	521.7	522.1	520.4	520.9	522.2	522.8	522.6	524.8
POL	7.1	1.9	7	6–8	521.5	0.7	2.5	521.4	521.2	520.9	521.0	521.2	521.1	520.9	521.7	521.8	521.5	523.4
CAN	5.8	2.2	8	7–8	520.6	0.8	2.8	519.5	519.9	521.4	521.4	520.1	519.9	519.9	521.0	521.1	520.5	522.2
DNK	5.9	2.0	9	9–10	518.4	0.8	2.3	518.1	518.2	519.4	519.4	517.6	518.1	517.5	519.4	518.9	517.1	518.6
SVN	7.1	1.5	10	10–12	515.2	0.8	2.3	516.4	516.0	514.3	514.9	514.7	515.3	515.7	514.3	514.1	515.2	515.9
BEL	8.3	2.6	11	9–11	517.2	0.7	2.3	516.1	516.7	516.9	517.2	517.4	518.1	517.0	516.7	516.7	517.6	518.4
CHE	8.2	2.5	12	11–12	514.5	0.5	1.5	514.2	514.8	514.0	514.4	514.9	515.2	514.8	513.9	513.7	515.2	514.0
DEU	9.6	2.5	13	13–13	509.8	1.0	3.1	509.1	509.1	509.2	509.2	511.4	511.5	510.4	509.8	509.4	509.8	508.4
FIN	8.9	3.0	14	14–16	506.7	1.0	3.7	506.9	506.5	506.7	507.3	506.5	506.9	506.8	508.0	507.9	506.0	504.3
IRL	5.8	1.3	15	**15–23**	502.2	2.6	**8.2**	505.2	504.8	501.6	502.1	499.9	500.7	500.0	505.1	504.9	497.1	502.5
CZE	9.2	2.0	16	**14–17**	505.1	1.0	3.7	504.9	504.3	503.4	504.2	505.3	505.8	505.8	505.1	505.0	505.5	507.1
GBR	8.2	2.5	17	**14–17**	505.6	1.0	3.3	503.9	504.9	506.6	504.6	507.2	505.7	505.0	505.8	505.8	506.6	505.4
NZL	8.1	2.2	18	**18–22**	502.4	1.2	4.0	503.3	504.3	502.5	502.0	501.8	501.6	501.6	502.4	504.8	501.9	500.8
FRA	**10.1** 1	3.0	19	**17–20**	502.8	0.9	2.4	502.1	502.2	502.5	503.0	503.9	503.9	503.9	501.8	501.5	503.1	503.3
AUT	8.4	1.8	20	**20–23**	500.8	0.9	2.7	500.9	501.7	500.1	499.4	501.9	500.7	501.4	499.6	500.4	501.0	502.1
PRT	**10.6** 1	2.8	21	**17–21**	501.9	1.2	3.8	500.1	500.1	500.4	501.2	502.4	502.7	502.5	502.4	502.0	502.6	503.9
LVA	6.4	1.7	22	**22–27**	496.8	1.7	**5.1**	499.7	498.6	497.2	497.7	494.6	495.3	495.1	497.9	497.9	494.7	496.5
NOR	**10.7** 1	**3.7**	23	**18–23**	501.8	1.5	4.2	499.4	499.8	502.0	502.0	503.7	503.4	503.4	500.6	500.3	502.7	502.0
AUS	7.3	2.4	24	24–26	495.7	0.9	3.7	495.3	496.3	497.8	495.6	496.0	495.5	495.5	495.6	495.7	495.6	494.0
SWE	**12.7** 1	**5.9**	25	**21–25**	498.4	3.3	**10.1** 1	491.8	493.4	499.1	499.7	501.3	501.1	501.3	497.9	498.0	501.9	496.8
ITA	**12.4** 1	2.8	26	25–27	492.0	2.3	**5.4**	490.4	490.1	489.4	490.1	494.7	494.4	494.0	490.1	489.9	494.6	494.1
ISL	9.7	**4.4**	27	**24–27**	494.2	2.8	**8.9**	489.1	491.3	496.5	498.0	495.1	495.6	495.8	495.9	495.2	493.3	490.5
LUX	**10.4** 1	2.8	28	28–28	486.5	0.9	2.6	486.8	486.5	485.5	486.3	487.2	487.4	486.6	485.3	485.2	487.7	487.5
LTU	7.4	1.5	29	**29–34**	482.0	1.7	**5.5**	485.5	484.4	482.1	483.0	480.1	480.9	480.6	482.0	481.9	480.6	480.9
RUS	7.9	2.2	30	29–31	483.7	0.7	2.1	484.6	484.2	483.6	484.6	482.9	483.7	483.9	484.0	483.7	482.5	482.5
SVK	8.0	1.8	31	**29–32**	483.2	0.6	2.4	484.5	483.9	482.8	483.4	482.8	483.3	483.0	483.2	483.1	482.1	482.7
HUN	8.6	2.0	32	**29–32**	483.2	0.7	2.4	484.1	483.8	483.7	483.4	482.9	482.7	482.9	484.0	483.7	482.6	481.6
ESP	**10.6** 1	2.9	33	**32–35**	481.5	1.7	**5.8**	482.4	482.4	481.7	482.5	481.6	482.1	482.3	482.4	481.9	480.4	476.7
USA	**4.0**	2.0	34	**29–36**	482.2	2.4	**6.6**	481.6	483.1	484.9	485.7	479.5	480.4	480.5	484.5	484.5	479.1	480.7
BLR	7.8	2.4	35	**32–36**	480.3	1.7	**5.4**	477.7	477.2	481.4	482.6	479.5	480.3	480.5	481.6	481.6	480.1	481.1
MLT	9.8	**3.9**	36	**33–37**	476.6	2.8	**9.2**	474.2	476.0	479.8	471.2	480.3	475.0	476.8	475.6	476.6	480.5	476.5
HRV	**11.8** 1	2.7	37	36–37	470.8	1.8	5.0	471.8	469.0	468.3	471.7	472.9	471.1	473.3	468.8	468.5	471.1	472.3
TUR	6.7	1.6	38	38–39	460.3	2.0	**6.2**	464.0	462.9	460.8	462.2	458.0	459.3	459.4	460.0	460.0	457.8	458.7
ISR	**12.1** 1	**4.5**	39	38–39	461.7	1.6	4.3	459.9	461.4	462.4	461.4	463.5	462.6	462.3	459.3	459.2	463.3	463.0
GRC	**10.7** 1	2.7	40	40–41	439.1	2.5	**9.2**	440.9	440.1	440.0	441.3	438.2	439.8	438.8	439.3	439.2	440.0	432.2
MYS	**1.2**	0.6	41	**41–44**	429.1	4.8	**12.5** 1	435.8	433.4	432.2	433.5	423.3	424.6	425.4	431.5	432.4	424.4	423.6
ALB	8.0	1.9	42	**41–44**	429.6	2.2	**6.3**	432.0	431.1	430.5	426.5	427.8	427.8	427.8	432.2	432.8	428.5	428.2
BRN	6.1	1.7	43	42–44	427.7	3.2	**8.1**	430.9	430.0	428.8	430.0	423.2	424.4	424.2	428.8	429.4	423.7	431.3
MNE	**17.3** 1	**3.8**	44	**40–44**	433.1	3.5	**10.7** 1	430.5	431.3	429.8	428.1	436.8	436.3	436.1	431.1	430.4	438.8	434.8
BIH	**18.8** 1	**3.9**	45	45–45	413.9	3.8	**10.5** 1	410.7	410.4	410.3	409.8	417.3	417.3	417.1	412.2	411.2	420.3	416.6

*Note.* %NA = proportion of item responses with missing data; %NR = proportion of item responses that are not reached; ${\mathrm{rk}}_{\mathrm{UW}}$ = country rank from model UW; ${\mathrm{rk}}_{\mathrm{Int}}$ = interval of country ranks obtained from 11 different scaling models; Aver = average of country means across 11 models; SD = standard deviation of country means across 11 models; rg = range of country means across 11 models; UW = scoring as wrong (Section 2.1); UP = MC items scored as partially correct (Section 2.2); UN1 = ignoring not reached items (Section 4.2.1); UN2 = including proportion of not reached items in background model (Section 4.2.1); UO1 = ignoring missing item responses (Section 2.3); MO2 = model-based latent ignorability (Section 2.4, Equations (10) and (11)); IO2 = imputed under latent ignorability (Section 2.4.1, Equations (10) and (11)); MM2 = Mislevy-Wu model with item format-specific δ parameters (Section 2.5, Equation (14)); IM2 = imputed under Mislevy-Wu model with item format specific d parameters (Section 2.5, Equation (14)); IF1 = FCS imputation based on item responses (Section 2.6 and Section 4.2.2); IF2 = FCS imputation based on item responses and response indicators (Section 2.6 and Section 4.2.2); The following entries in the table are printed in bold: Missing proportions (%NA) larger than 10.0% and smaller than 5.0%, not reached proportions larger than 3.0%, country rank differences larger than 2, ranges in country means larger than 5.0. See Appendix B for country labels.

## Data Availability

The PISA 2018 dataset is available from https://www.oecd.org/pisa/data/2018database/ (accessed on 15 April 2021).

## Abbreviations

The following abbreviations are used in this manuscript:

1PL	one-parameter logistic model
2PL	two-parameter logistic model
AIC	Akaike information criterion
BIC	Bayesian information criterion
CR	constructed-response
DIF	differential item functioning
FCS	fully conditional specification
GHP	Gilula–Haberman penalty
IRT	item response theory
LBM	latent background model
LSA	large-scale assessment
M	mean
MAR	missing at random
MC	multiple-choice
MST	multi-stage testing
PISA	programme for international student assessment
PLS	partial least squares
PMM	predictive mean matching
RMSE	root mean square error
SD	standard deviation

## Appendix A. Additional Information for the Illustrative Simulation Study

In Table A1, the computed β parameter used in the illustrative simulation study as a function of the proportion of missing data and the missingness parameter δ is shown.

**Table A1:** Computed β parameter in the Mislevy-Wu model as a function of the proportion of missing data and the missingness parameter δ.

Miss %	δ				
	−10	−3	−2	−1	0
5%	−3.728	−3.805	−3.906	−4.100	−4.422
10%	−2.548	−2.678	−2.821	−3.061	−3.417
20%	−1.001	−1.288	−1.513	−1.827	−2.228
30%	−0.322	−0.255	−0.568	−0.947	−1.384

*Note.* Miss% = proportion of missing data.

In Table A2, the RMSE for the estimated mean and the standard deviation is shown for the different missing data treatments as a function of the proportion of missing data and the missingness parameter δ.

**Table A2:** Root mean square error (RMSE) for the mean and the standard deviation for different missing data treatments as a function of the missing proportion and the missingness parameter δ.

δ	Mean					Standard Deviation				
	−10	−3	−2	−1	0	−10	−3	−2	−1	0
Model										
	*5% missing data*									
CD	0.049	0.054	0.054	0.055	0.049	0.047	0.053	0.054	0.053	0.049
MM1	0.051	0.049	0.055	0.054	0.061	0.048	0.049	0.053	0.050	0.056
UW	0.049	0.052	0.053	0.067	0.087	0.047	0.050	0.046	0.050	0.054
UO	0.110	0.105	0.102	0.082	0.058	0.077	0.075	0.073	0.065	0.055
MO2	0.106	0.099	0.091	0.071	0.053	0.077	0.072	0.067	0.059	0.052
IF1	0.109	0.106	0.102	0.082	0.058	0.075	0.074	0.074	0.063	0.054
IF2	0.108	0.103	0.097	0.078	0.053	0.074	0.074	0.070	0.063	0.052
	*10% missing data*									
CD	0.052	0.052	0.050	0.051	0.056	0.050	0.053	0.049	0.050	0.055
MM1	0.053	0.056	0.060	0.059	0.063	0.051	0.055	0.056	0.053	0.054
UW	0.052	0.058	0.076	0.100	0.149	0.050	0.053	0.054	0.052	0.048
UO	0.168	0.144	0.125	0.111	0.066	0.107	0.082	0.073	0.079	0.060
MO2	0.159	0.132	0.119	0.096	0.054	0.097	0.072	0.074	0.071	0.053
IF1	0.169	0.148	0.128	0.112	0.067	0.106	0.085	0.074	0.078	0.060
IF2	0.161	0.142	0.125	0.104	0.057	0.097	0.080	0.076	0.076	0.056
	*20% missing data*									
CD	0.052	0.052	0.051	0.049	0.051	0.052	0.050	0.051	0.048	0.050
MM1	0.077	0.058	0.064	0.069	0.081	0.070	0.053	0.058	0.058	0.061
UW	0.052	0.093	0.139	0.208	0.278	0.051	0.056	0.046	0.056	0.061
UO	0.211	0.219	0.193	0.154	0.091	0.158	0.144	0.118	0.092	0.075
MO2	0.211	0.218	0.187	0.129	0.053	0.158	0.141	0.112	0.080	0.050
IF1	0.216	0.223	0.192	0.157	0.086	0.159	0.145	0.112	0.092	0.068
IF2	0.222	0.220	0.194	0.140	0.056	0.166	0.138	0.114	0.087	0.054
	*30% missing data*									
CD	0.053	0.051	0.052	0.053	0.055	0.053	0.051	0.051	0.052	0.054
MM1	0.128	0.063	0.072	0.083	0.092	0.159	0.057	0.059	0.064	0.064
UW	0.051	0.167	0.232	0.306	0.372	0.051	0.049	0.049	0.056	0.061
UO	0.211	0.247	0.234	0.190	0.095	0.222	0.179	0.148	0.117	0.076
MO2	0.207	0.247	0.238	0.171	0.060	0.225	0.179	0.152	0.107	0.057
IF1	0.220	0.253	0.240	0.193	0.098	0.221	0.180	0.149	0.115	0.075
IF2	0.216	0.256	0.242	0.179	0.064	0.224	0.180	0.149	0.109	0.061

*Note.* CD = complete-data analysis; UW = scoring as wrong (Section 2.1); MM1 = Mislevy-Wu model with common d parameter (Section 2.5, Equation (14)); UO = ignoring missing item responses (Section 2.3); MO2 = model-based latent ignorability (Section 2.4, Equations (10) and (11)); IF1 = FCS imputation based on item responses (Section 2.6); IF2 = FCS imputation based on item responses and response indicators (Section 2.6).

## Appendix B. Country Labels Used in the PISA 2018 Mathematics Case Study

The country labels used in Table 2, Table 5, Table 6 and Table 7, Table A3, Table A4 and Table A5 are as follows: ALB = Albania; AUS = Australia; AUT = Austria; BEL = Belgium; BIH = Bosnia and Herzegovina; BLR = Belarus; BRN = Brunei Darussalam; CAN = Canada; CHE = Switzerland; CZE = Czech Republic; DEU = Germany; DNK = Denmark; ESP = Spain; EST = Estonia; FIN = Finland; FRA = France; GBR = United Kingdom; GRC = Greece; HKG = Hong Kong; HRV = Croatia; HUN = Hungary; IRL = Ireland; ISL = Iceland; ISR = Israel; ITA = Italy; JPN = Japan; KOR = Korea; LTU = Lithuania; LUX = Luxembourg; LVA = Latvia; MLT = Malta; MNE = Montenegro; MYS = Malaysia; NLD = Netherlands; NOR = Norway; NZL = New Zealand; POL = Poland; PRT = Portugal; RUS = Russian Federation; SGP = Singapore; SVK = Slovak Republic; SVN = Slovenia; SWE = Sweden; TUR = Turkey; USA = United States.

## Appendix C. Further Results of the PISA 2018 Mathematics Case Study

In Table A3, standard errors for country means for the PISA 2018 mathematics case study resulting from 11 different scaling models are shown.

In Table A4, standard errors for country mean differences between the models UW, MO2 and MM2 are presented.

In Table A5, country standard deviations for the PISA 2018 mathematics case study resulting from 11 different scaling models are reported.

**Table A3:** Standard errors for country means for PISA 2018 mathematics from 11 different scaling models for missing item responses.

Country	Aver	SD	rg	UW	UP	UN1	UN2	UO1	MO2	IO2	MM2	IM2	IF1	IF2
ALB	2.02	0.06	0.24	2.07	2.04	2.03	2.16	1.99	2.02	2.02	2.02	2.02	1.99	1.91
AUS	1.69	0.03	0.11	1.73	1.71	1.67	1.75	1.64	1.68	1.68	1.70	1.69	1.68	1.69
AUT	2.53	0.05	0.14	2.56	2.55	2.54	2.60	2.47	2.53	2.49	2.61	2.55	2.47	2.47
BEL	1.79	0.06	0.16	1.85	1.84	1.84	1.83	1.71	1.78	1.71	1.86	1.82	1.70	1.75
BIH	2.37	0.19	0.56	2.53	2.53	2.54	2.52	2.26	2.32	2.27	2.51	2.48	2.13	1.99
BLR	2.29	0.02	0.06	2.31	2.32	2.31	2.28	2.26	2.27	2.27	2.30	2.29	2.27	2.26
BRN	1.56	0.02	0.06	1.58	1.57	1.52	1.53	1.55	1.56	1.58	1.56	1.55	1.57	1.56
CAN	2.05	0.03	0.12	2.05	2.05	2.06	2.05	2.02	2.03	2.03	2.04	2.03	2.05	2.14
CHE	2.28	0.02	0.05	2.28	2.29	2.27	2.26	2.25	2.27	2.26	2.30	2.29	2.27	2.28
CZE	2.15	0.02	0.05	2.16	2.17	2.17	2.16	2.13	2.15	2.13	2.17	2.14	2.13	2.17
DEU	2.51	0.07	0.22	2.56	2.56	2.58	2.59	2.46	2.49	2.48	2.56	2.56	2.43	2.38
DNK	1.82	0.02	0.08	1.86	1.83	1.81	1.84	1.78	1.81	1.81	1.83	1.83	1.84	1.79
ESP	1.21	0.03	0.10	1.23	1.23	1.24	1.24	1.15	1.19	1.17	1.24	1.21	1.17	1.20
EST	1.80	0.02	0.06	1.78	1.78	1.82	1.80	1.82	1.80	1.80	1.78	1.79	1.83	1.81
FIN	1.97	0.02	0.07	1.98	1.98	1.96	1.95	1.96	2.00	1.98	1.98	1.95	1.95	1.93
FRA	2.17	0.03	0.09	2.20	2.19	2.21	2.20	2.14	2.15	2.14	2.20	2.21	2.12	2.13
GBR	2.53	0.05	0.18	2.58	2.57	2.52	2.65	2.47	2.48	2.48	2.52	2.53	2.51	2.53
GRC	2.68	0.07	0.26	2.69	2.70	2.75	2.73	2.68	2.69	2.68	2.72	2.72	2.61	2.49
HKG	2.83	0.02	0.05	2.83	2.85	2.82	2.84	2.81	2.82	2.80	2.85	2.84	2.82	2.85
HRV	2.36	0.09	0.25	2.41	2.45	2.46	2.42	2.29	2.33	2.29	2.43	2.45	2.26	2.21
HUN	2.26	0.06	0.16	2.33	2.32	2.26	2.31	2.19	2.26	2.23	2.32	2.28	2.21	2.17
IRL	2.01	0.01	0.05	2.00	2.01	2.04	2.02	2.01	2.03	2.01	2.00	2.00	1.99	2.01
ISL	2.06	0.04	0.11	2.03	2.01	2.10	2.10	2.11	2.07	2.11	2.00	2.02	2.08	2.01
ISR	3.65	0.18	0.56	3.84	3.73	3.66	3.80	3.52	3.65	3.62	3.82	3.80	3.40	3.27
ITA	2.47	0.06	0.14	2.51	2.53	2.52	2.50	2.39	2.43	2.40	2.53	2.51	2.42	2.39
JPN	2.42	0.01	0.04	2.42	2.44	2.42	2.41	2.40	2.43	2.41	2.43	2.41	2.40	2.42
KOR	2.89	0.09	0.33	2.94	2.93	2.85	3.12	2.79	2.86	2.86	2.90	2.91	2.79	2.88
LTU	1.91	0.02	0.07	1.89	1.91	1.89	1.88	1.93	1.93	1.92	1.91	1.90	1.93	1.95
LUX	1.72	0.03	0.09	1.72	1.72	1.76	1.74	1.69	1.71	1.70	1.75	1.75	1.67	1.70
LVA	1.90	0.02	0.05	1.92	1.93	1.89	1.90	1.88	1.89	1.89	1.89	1.91	1.88	1.89
MLT	2.75	0.21	0.82	2.91	2.81	2.69	3.22	2.63	2.74	2.71	2.81	2.78	2.51	2.40
MNE	1.31	0.07	0.23	1.36	1.35	1.32	1.40	1.27	1.33	1.27	1.37	1.34	1.21	1.17
MYS	2.66	0.09	0.30	2.57	2.62	2.59	2.57	2.71	2.72	2.69	2.63	2.59	2.73	2.87
NLD	2.22	0.03	0.10	2.26	2.25	2.16	2.26	2.17	2.21	2.22	2.23	2.23	2.19	2.26
NOR	1.65	0.06	0.17	1.71	1.68	1.65	1.68	1.61	1.65	1.64	1.69	1.69	1.56	1.54
NZL	1.81	0.05	0.16	1.86	1.84	1.79	1.90	1.78	1.81	1.81	1.85	1.82	1.77	1.73
POL	2.66	0.04	0.12	2.66	2.67	2.71	2.71	2.65	2.68	2.68	2.69	2.66	2.61	2.58
PRT	2.27	0.04	0.13	2.29	2.30	2.34	2.31	2.22	2.25	2.23	2.28	2.28	2.22	2.21
RUS	2.63	0.02	0.07	2.62	2.62	2.68	2.64	2.61	2.61	2.63	2.62	2.63	2.65	2.61
SGP	1.56	0.05	0.14	1.53	1.54	1.52	1.52	1.60	1.55	1.56	1.52	1.53	1.63	1.66
SVK	2.40	0.03	0.08	2.37	2.39	2.40	2.38	2.44	2.41	2.45	2.38	2.40	2.38	2.38
SVN	1.99	0.03	0.08	2.00	2.00	1.98	1.98	1.95	1.98	1.97	2.03	2.01	1.95	2.01
SWE	2.52	0.05	0.16	2.50	2.48	2.57	2.55	2.55	2.55	2.57	2.54	2.55	2.48	2.41
TUR	1.93	0.03	0.10	1.89	1.90	1.91	1.89	1.95	1.94	1.95	1.91	1.92	1.96	1.98
USA	2.77	0.04	0.14	2.83	2.82	2.71	2.69	2.77	2.80	2.78	2.78	2.76	2.82	2.74

*Note.* Aver = average of standard errors of country means across 11 models; SD = standard deviation of standard errors of country means across 11 models; rg = range of standard errors of country means across 11 models; UW = scoring as wrong (Section 2.1); UP = MC items scored as partially correct (Section 2.2); UN1 = ignoring not reached items (Section 4.2.1); UN2 = including proportion of not reached items in background model (Section 4.2.1); UO1 = ignoring missing item responses (Section 2.3); MO2 = model-based latent ignorability (Section 2.4, Equations (10) and (11)); IO2 = imputed under latent ignorability (Section 2.4.1, Equations (10) and (11)); MM2 = Mislevy-Wu model with item format specific d parameters (Section 2.5, Equation (14)); IM2 = imputed under Mislevy-Wu model with item format specific d parameters (Section 2.5, Equation (14)); IF1 = FCS imputation based on item responses (Section 2.6 and Section 4.2.2); IF2 = FCS imputation based on item responses and response indicators (Section 2.6 and Section 4.2.2); See Appendix B for country labels.

**Table A4:** Standard errors for country mean differences between three different models UW, MO2 and MM2 for PISA 2018 mathematics.

Country	−UW−	−MO2−	−MM2−	UW–MO2	UW–MM2	MO2–MM2
ALB	2.068	2.025	2.022	0.044	0.058	0.014
AUS	1.732	1.678	1.701	0.031	0.030	0.001
AUT	2.562	2.530	2.607	0.028	0.009	0.036
BEL	1.847	1.782	1.863	0.013	0.007	0.019
BIH	2.533	2.316	2.513	0.117	0.019	0.093
BLR	2.311	2.274	2.303	0.028	0.010	0.037
BRN	1.580	1.562	1.560	0.014	0.004	0.018
CAN	2.046	2.032	2.044	0.002	0.020	0.018
CHE	2.283	2.270	2.299	0.004	0.017	0.013
CZE	2.159	2.148	2.173	0.039	0.024	0.014
DEU	2.564	2.489	2.560	0.046	0.011	0.035
DNK	1.856	1.806	1.827	0.008	0.021	0.013
ESP	1.232	1.185	1.238	0.016	0.003	0.013
EST	1.776	1.799	1.783	0.026	0.008	0.018
FIN	1.984	2.001	1.981	0.051	0.018	0.032
FRA	2.197	2.154	2.201	0.024	0.009	0.033
GBR	2.580	2.484	2.522	0.079	0.056	0.023
GRC	2.686	2.686	2.720	0.046	0.035	0.011
HKG	2.829	2.820	2.851	0.004	0.012	0.008
HRV	2.413	2.333	2.430	0.015	0.031	0.045
HUN	2.328	2.263	2.316	0.017	0.015	0.001
IRL	1.997	2.030	2.001	0.036	0.004	0.040
ISL	2.028	2.070	2.002	0.000	0.036	0.037
ISR	3.835	3.652	3.816	0.106	0.019	0.084
ITA	2.512	2.434	2.527	0.037	0.014	0.050
JPN	2.416	2.427	2.430	0.037	0.002	0.034
KOR	2.944	2.865	2.896	0.075	0.045	0.030
LTU	1.891	1.926	1.910	0.039	0.027	0.013
LUX	1.722	1.706	1.746	0.004	0.016	0.012
LVA	1.920	1.893	1.892	0.021	0.003	0.018
MLT	2.912	2.740	2.811	0.145	0.096	0.048
MNE	1.364	1.334	1.375	0.056	0.018	0.038
MYS	2.570	2.717	2.632	0.149	0.061	0.088
NLD	2.257	2.213	2.226	0.009	0.014	0.005
NOR	1.711	1.650	1.692	0.034	0.010	0.023
NZL	1.858	1.814	1.853	0.007	0.013	0.005
POL	2.664	2.681	2.685	0.023	0.000	0.022
PRT	2.288	2.247	2.285	0.018	0.011	0.006
RUS	2.617	2.607	2.620	0.011	0.007	0.004
SGP	1.530	1.549	1.522	0.003	0.011	0.008
SVK	2.374	2.415	2.380	0.022	0.011	0.011
SVN	1.999	1.982	2.031	0.007	0.018	0.025
SWE	2.505	2.551	2.541	0.042	0.026	0.016
TUR	1.890	1.940	1.907	0.064	0.030	0.034
USA	2.834	2.801	2.780	0.089	0.015	0.071

*Note.* Aver = average of standard errors of country means across 11 models; SD = standard deviation of standard errors of country means across 11 models; rg = range of standard errors of country means across 11 models; UW = scoring as wrong (Section 2.1); MO2 = model-based latent ignorability (Section 2.4, Equations (10) and (11)); MM2 = Mislevy-Wu model with item format specific d parameters (Section 2.5, Equation (14)); See Appendix B for country labels.

**Table A5:** Country standard deviations for PISA 2018 mathematics from 11 different scaling models for missing item responses.

Country	Aver	SD	rg	UW	UP	UN1	UN2	UO1	MO2	IO2	MM2	IM2	IF1	IF2
ALB	69.6	1.0	3.7	70.9	69.8	69.1	71.5	69.4	69.4	69.3	68.9	69.5	70.0	67.9
AUS	91.4	0.9	2.3	92.4	91.9	90.5	91.0	90.7	90.8	90.9	90.9	90.9	92.6	92.9
AUT	90.5	0.6	1.6	90.9	90.8	89.9	90.9	89.7	89.9	90.3	91.2	91.1	90.0	91.1
BEL	88.4	0.7	2.4	88.6	88.6	88.5	87.8	87.5	87.9	87.8	88.9	88.7	88.5	89.9
BIH	81.7	3.5	11.4	84.9	84.4	83.9	84.2	80.5	81.0	80.9	84.2	83.2	78.0	73.5
BLR	90.6	1.0	2.9	90.1	90.4	90.3	89.1	91.6	91.1	91.2	89.7	89.8	91.9	92.0
BRN	90.0	1.0	3.0	89.5	89.3	89.5	88.5	90.9	90.3	90.8	89.2	89.5	91.5	91.1
CAN	86.7	1.6	5.9	86.5	86.3	85.5	85.1	86.5	86.4	86.5	85.6	85.8	88.2	91.0
CHE	88.1	0.3	1.0	87.7	88.2	88.7	87.7	88.0	87.8	87.7	88.3	88.0	88.6	88.2
CZE	90.1	0.8	2.7	88.7	89.3	90.4	89.6	90.9	90.3	90.1	89.7	89.8	90.6	91.4
DEU	91.3	1.2	3.6	92.6	92.2	92.0	92.4	90.5	90.9	90.9	92.2	92.3	89.4	89.0
DNK	83.8	0.8	2.4	83.9	83.7	83.1	82.9	83.8	83.6	83.9	83.0	83.2	85.0	85.3
ESP	83.1	0.8	2.7	83.2	83.1	83.9	83.0	82.1	82.1	82.3	83.0	83.3	83.1	84.7
EST	83.5	1.0	3.0	82.6	82.6	83.4	82.5	84.1	83.8	83.6	83.0	82.6	85.1	85.5
FIN	84.4	0.9	2.6	83.0	83.2	85.4	84.6	85.6	85.2	85.2	83.8	83.8	85.0	83.7
FRA	91.1	0.7	1.8	91.6	91.5	91.8	91.1	90.5	90.6	90.2	92.0	92.1	90.9	90.3
GBR	92.2	1.3	3.9	94.1	93.3	91.1	94.0	90.2	91.2	91.0	92.1	92.2	91.7	92.8
GRC	83.0	1.3	4.8	82.2	82.6	84.2	83.5	84.1	83.6	83.6	83.3	83.4	83.5	79.4
HKG	89.6	1.1	4.0	89.1	89.5	88.3	88.9	89.2	89.2	89.3	89.4	89.5	90.5	92.3
HRV	82.5	1.6	5.3	82.8	83.9	84.6	82.8	81.5	82.3	81.2	83.9	84.1	81.1	79.3
HUN	92.0	1.1	4.1	93.1	92.9	92.4	92.2	92.0	92.4	92.0	92.5	92.4	91.0	88.9
IRL	77.8	0.7	1.7	77.1	77.4	78.3	77.6	78.4	78.4	78.3	76.9	76.9	78.2	78.6
ISL	86.2	1.2	4.1	87.0	86.1	87.1	86.4	87.6	87.1	87.3	85.5	85.7	85.2	83.6
ISR	109.2	2.7	9.4	112.0	109.7	110.0	110.4	108.4	109.0	109.2	111.5	111.8	106.2	102.6
ITA	92.1	0.9	2.4	92.3	92.7	93.2	92.6	90.8	91.0	91.2	92.8	93.1	90.9	92.0
JPN	85.2	0.8	2.2	84.4	85.0	84.8	84.2	85.8	85.7	85.9	84.5	84.6	86.2	86.4
KOR	91.2	1.4	4.9	92.8	92.2	89.9	94.0	89.1	90.4	90.6	91.3	91.5	89.7	91.4
LTU	90.3	0.8	2.8	88.8	89.7	90.4	89.5	91.2	90.6	90.7	90.0	90.2	91.6	91.1
LUX	92.9	0.6	2.0	92.6	92.6	93.7	92.7	92.9	92.8	93.3	93.5	93.8	92.4	91.7
LVA	80.4	0.7	2.2	79.8	80.4	80.2	79.4	80.9	80.7	80.4	79.9	80.2	81.6	81.3
MLT	99.0	5.4	18.2	105.2	101.4	98.2	105.8	96.7	100.0	99.4	101.7	101.0	92.1	87.6
MNE	79.7	2.8	9.3	82.4	82.3	80.9	81.8	78.3	79.1	78.7	81.3	81.0	77.3	73.1
MYS	89.0	3.1	10.2	85.8	87.6	86.5	85.9	90.5	90.7	89.7	87.8	86.7	91.6	95.9
NLD	89.8	0.9	3.0	89.9	90.2	88.9	88.9	89.7	89.6	89.4	89.4	89.1	90.8	91.8
NOR	89.7	1.2	4.1	91.0	90.0	90.8	90.4	89.1	89.2	89.3	90.4	90.7	88.6	86.8
NZL	93.2	0.4	1.4	93.1	92.7	93.0	93.9	93.1	93.4	93.4	93.7	92.5	93.7	93.0
POL	88.6	0.3	0.9	88.3	88.5	88.6	88.4	88.9	89.0	89.2	88.3	88.4	88.6	88.7
PRT	95.2	0.5	2.1	94.4	95.1	96.4	95.3	95.5	95.1	95.5	94.8	95.2	95.4	94.7
RUS	79.9	0.4	1.6	79.4	79.7	80.3	79.2	80.2	79.8	80.1	79.6	79.6	80.8	80.1
SGP	91.7	1.9	6.7	91.5	91.8	89.7	89.9	91.6	91.3	91.3	90.8	90.8	93.8	96.4
SVK	91.9	0.5	1.4	91.4	91.7	92.2	91.1	92.5	92.3	92.4	91.9	92.0	92.1	91.2
SVN	86.0	0.5	1.6	85.7	86.1	86.3	85.5	85.5	85.3	85.7	86.4	86.3	86.4	87.0
SWE	88.5	1.2	4.2	87.6	86.8	90.3	89.3	89.5	89.1	89.4	88.6	88.8	88.5	86.1
TUR	90.9	2.0	5.7	88.5	89.3	89.8	88.7	92.4	91.5	91.7	89.9	90.0	94.0	94.2
USA	95.3	2.0	5.9	93.5	93.9	94.0	92.7	97.2	96.4	96.7	94.0	94.0	98.6	97.4

*Note.* Aver = average of country standard deviations across 11 models; SD = standard deviation of country standard deviations across 11 models; rg = range of country standard deviations across 11 models; UW = scoring as wrong (Section 2.1); UP = MC items scored as partially correct (Section 2.2); UN1 = ignoring not reached items (Section 4.2.1); UN2 = including proportion of not reached items in background model (Section 4.2.1); UO1 = ignoring missing item responses (Section 2.3); MO2 = model-based latent ignorability (Section 2.4, Equations (10) and (11)); IO2 = imputed under latent ignorability (Section 2.4.1, Equations (10) and (11)); MM2 = Mislevy-Wu model with item format specific d parameters (Section 2.5, Equation (14)); IM2 = imputed under Mislevy-Wu model with item format specific d parameters (Section 2.5, Equation (14)); IF1 = FCS imputation based on item responses (Section 2.6 and Section 4.2.2); IF2 = FCS imputation based on item responses and response indicators (Section 2.6 and Section 4.2.2); See Appendix B for country labels.
